# Measuring Methane Emissions in Ambient Air with a Low-Cost, Portable Sensor System: Focus on Scalability and Transferability of the Model

**DOI:** 10.3390/s26134321

**Published:** 2026-07-07

**Authors:** Lorenzo Bertin, Matteo Mentasti, Fabrizio Pittorino, Veronica Villa, Emanuele Zanni, Gabriele Viscardi, Yuri Ponzani, Andrea Massara, Manuel Roveri, Raffaele Dellaca’, Laura Capelli

**Affiliations:** 1 Department of Chemistry, Materials and Chemical Engineering “Giulio Natta”, Politecnico di Milano, Piazza Leonardo da Vinci 32, 20133 Milan, Italy; veronica.villa@polimi.it (V.V.); laura.capelli@polimi.it (L.C.); 2 Department of Electronics, Information, and Bioengineering, Politecnico di Milano, via Giuseppe Ponzio 34, 20133 Milan, Italy; matteo.mentasti@polimi.it (M.M.); fabrizio.pittorino@polimi.it (F.P.); emanuele.zanni@polimi.it (E.Z.); gabriele.viscardi@polimi.it (G.V.); manuel.roveri@polimi.it (M.R.); raffaele.dellaca@polimi.it (R.D.); 3 Recycle2Trade Ltd., Science Park Square, Brighton BN1 9SB, UK; info@recycle2trade.com; 4 Integraciones Digitales Gold SL (Indigo), 8820 Barcelona, Spain; andrea@adaptivecity.com

**Keywords:** low-cost gas sensors, methane emissions, landfill monitoring, MOX sensors, machine learning, calibration transfer, sensor toolbox, walkover survey

## Abstract

Landfills represent a significant source of methane emissions, with important environmental, climatic and safety impacts due to the widespread and variable nature of these emissions. Traditional monitoring methods, such as flow chambers coupled with flame ionisation detectors (FIDs), provide high accuracy but are limited in terms of spatial representativeness, operational flexibility and cost, especially during large-scale or continuous monitoring campaigns. Within this context, the European ESCAPE project aims to develop a low-cost, portable and modular platform for the detection and quantification of low methane concentrations in ambient air at complex environmental sites. The system is based on commercial MOX and NDIR sensors integrated into portable toolboxes equipped with dedicated chambers, regulated suction systems and autonomous data acquisition units with real-time transmission. This work describes the development and testing of two identical toolboxes to assess system reproducibility and the transferability of predictive models between devices. Laboratory and field tests were carried out under controlled and real landfill conditions, with comparisons against portable FID measurements. Results showed good agreement between predicted methane concentrations and reference data, with correlation indexes up to 0.77. Moreover, transferring the machine learning model between toolboxes did not produce statistically significant performance reductions, demonstrating promising robustness and generalizability of the proposed calibration strategy.

## 1. Introduction

Methane (CH_4_) is the second most significant anthropogenic greenhouse gas after carbon dioxide [1], with a global warming potential approximately 28 times greater than that of CO_2_ over a 100-year time horizon [2]. Methane emissions account for approximately 11% of global anthropogenic greenhouse gas emissions [3]. Among the main anthropogenic sources, municipal solid waste landfills constitute a particularly significant source: the waste sector is the second-largest emitter of methane in Europe, with approximately 97 Mt CO_2_eq emitted in 2022 [4], of which landfills are responsible for around 80% [5]. At these sites, methane is produced by the anaerobic decomposition of the organic fraction of the waste and can be emitted diffusely across the landfill surface or as a point source through cracks, malfunctioning collection wells or areas with damaged capping [6,7]. The spatially heterogeneous and temporally variable nature of these emissions makes their monitoring particularly complex [8].

Accurate monitoring of landfill methane emissions is essential for both environmental and climate assessment and for safety reasons, as methane can form explosive mixtures with air at concentrations between 5% and 15% by volume [9]. Traditional measurement methods include static and dynamic flow chambers, which allow for accurate point measurements but are limited in terms of spatial representativeness and require long measurement times [10]. Optical techniques such as Differential Absorption Lidar (DIAL) allow for integrated measurements over large areas [11,12], while selective analytical techniques such as gas chromatography and the innovative plasma electron spectroscopy (PLES), which has recently shown remarkable potential for the precise analysis of methane and its gaseous impurities, enabling accurate quantification of methane and of the co-occurring trace species [13,14]; however, all these approaches are far from low-cost and require dedicated, expensive instrumentation and highly specialised personnel, which makes their direct on-site use by landfill operators impractical.

Walkovers using flame ionisation detectors (FIDs) are commonly used to identify emission hotspots and guide maintenance interventions, offering high accuracy but with relatively high costs and limited operability for extended campaigns [15]. These limitations highlight the need for complementary approaches capable of ensuring greater spatial coverage with reduced costs and operational complexity [16].

Low-cost gas sensors have opened up new possibilities for distributed and portable methane monitoring [17]. In particular, metal oxide semiconductor (MOX) sensors are of interest due to their high sensitivity and low cost, but they present challenges related to selectivity, drift over time, and sensitivity to environmental factors such as relative humidity [18]. NDIR sensors, on the other hand, are inherently more selective but generally have higher detection thresholds and are more expensive [19]. The integration of multi-sensor arrays with machine learning techniques can help overcome the limitations of individual sensors, combining information from different transduction principles to obtain more accurate and robust estimates of methane concentration [18,19].

The European ESCAPE (Environmental Sites CH_4_ Assessment Platform Europe) project, funded under the Eureka Eurostars programme (project no. 2204) [20], aims to develop a portable, low-cost “Sensor Toolbox” for monitoring methane emissions at complex environmental sites, such as landfills, integrating commercial MOX and NDIR sensors into a compact, autonomous system, capable of transmitting data in real time to a mobile application for georeferenced visualisation of the measurements.

A fundamental requirement for the practical application of the system is scalability. In this context, scalability does not merely mean reducing the cost of the individual device but also demonstrating that multiple units produced using the same architecture can provide sufficiently consistent results to allow the use of shared calibration strategies. The ability to transfer a calibration model from one toolbox to another is essential to limit the need to fully calibrate each unit individually, which would prevent the system’s practical applicability on a large scale.

For this reason, in this study, we decided to consider two replicated toolbox units (designated as V2 and V3), with the purpose of making some preliminary assessments regarding calibration transfer between devices. These toolboxes were manufactured at the same time, using the same selected sensors, the same electronic and pneumatic components, the same mechanical architecture and the same acquisition logic. The aim is therefore not to compare the performance of different versions of the system, but to verify whether two nominally identical devices can exhibit behaviour that is sufficiently reproducible to support the development of a transferable calibration approach.

The experimental workflow was organised as to include both a laboratory calibration phase and field training and testing campaigns phase. This approach was adopted initially to characterise, under controlled laboratory conditions, the response of the toolboxes to known concentrations of methane, interferents, and relative humidity, providing a useful experimental basis for validating and interpreting the field data. Field campaigns are essential to expose the sensors to the actual variability of operating conditions, including the spatial variability of emissions, the presence of interfering compounds, varying weather conditions, and site-specific characteristics. The field tests were carried out at two different Italian landfill sites and on days characterised by different weather conditions to increase the variability of the data collected and make the dataset more representative of the conditions encountered during monitoring.

## 2. Materials and Methods

### 2.1. Sensor Toolbox Design

The sensor toolbox was designed with the aim of realizing a portable, low-cost and easily replicable system. The design process involved selecting sensors through preliminary experimental screening, defining the chamber architecture, developing the control electronics and integrating them into a compact housing.

#### 2.1.1. Sensor Selection

The selection of sensors was carried out in two stages in a previous work by Villa et al. [21]. Initially, a market analysis was conducted to identify commercial MOX and NDIR sensors potentially suitable for detecting methane in ambient air. Subsequently, the candidate sensors were tested experimentally using a dedicated setup, evaluating their sensitivity to methane, response time, repeatability, sensitivity to relative humidity and cost [21]. This screening identified MOX sensors as the most suitable low-cost technology for methane detection, thanks to their sensitivity at low concentration (i.e., few ppm). In particular, the two TGS 2611 variants were proven to be the most sensitive to methane, as declared by the manufacturer and as reported also in previous work by other authors [22]. Based on these evaluations, the following sensors were selected for integration into the toolbox.

The TGS2611-E00 sensor (Figaro Engineering Inc., Osaka, Japan) is a MOX sensor with an analogue output, specifically designed for methane detection. Besides having a sensitive layer with increased selectivity to methane, the sensor also integrates an integrated activated carbon filter to further reduce sensitivity to compounds other than methane [23].

The TGS2611-C00 sensor (Figaro Engineering Inc., Osaka, Japan) is the version without the activated carbon filter. Thus, despite having an increased selectivity to methane given by the active sensing material, the sensor also responds to a certain extent to other volatile organic compounds. The differential responses of the two TGS2611 sensors (E-00 vs C-00) can be used to obtain information about the presence of interferents in the sampled air [23].

The SGP40 sensor (Sensirion AG, Stäfa, Switzerland) is a digital MOX sensor for volatile organic compounds (VOCs), with integrated compensation for temperature and relative humidity. Although not specific to methane, this sensor provides complementary information on air quality and the presence of other organic compounds [23,24].

The MH-441D sensor (Winsen Electronics Technology Co., Zhengzhou, China) is an NDIR sensor for methane with a digital output. This sensor offers greater selectivity than MOX sensors but has a higher detection threshold, i.e., approximately 1000 ppm according to the manufacturer’s datasheet [19]. For this reason, it was not primarily intended for low-ppm methane detection but rather as a supporting sensor for comparison with the MOX array at high methane concentrations and as an internal alarm indicator in the presence of high-concentration events.

It is well known that MOX sensors are intrinsically non-selective and retain a residual cross-sensitivity towards other reducing compounds and interferents. This also applies to the TGS2611-E00 which, despite being the variant least affected by interferents and marketed as “methane-specific”, still exhibits a non-negligible response to some of other compounds. In the specific context of landfill monitoring, the biogas can contain various trace species besides methane, particularly during the different stages of waste degradation; nevertheless, landfill gas is largely dominated by CH_4_ and CO_2_, which together typically account for more than 90% of its composition [1], so these two gases were taken as the main target of the present work. The residual cross-sensitivity intrinsic to MOX sensors is addressed by adopting a multi-sensor architecture coupled with machine learning models: this sensor fusion approach has the potential to improve the robustness of the methane prediction [22]. It should finally be stressed that, for the intended application, the goal is not a very high quantification accuracy but rather the capability to detect methane at low concentrations in ambient air (in the order of 10 ppm), which can be achieved neither with NDIR sensors nor with conventional low-cost gas leak detectors.

Table 1 reports the costs and specifications of the selected sensors.

#### 2.1.2. Sensor Toolbox Architecture

Two functional units of the ESCAPE Sensor Toolbox (designated as V2 and V3, respectively) were designed and realized for this study, built identically to the same specifications and using components from the same production batch, to enhance performance comparability and measurement reproducibility.

Each device consists of a PTFE measurement chamber with a volume of 23.5 mL (approximately 17.5 mL net of the sensors’ footprint), through which a continuous air flow, regulated by a flow damper, enables real-time sampling and analysis. The chamber houses the entire set of sensors described in Section 2.1.1: the two Figaro TGS2611 analogue MOX sensors (versions C-00 and E-00), the Sensirion SGP40 digital MOX sensor, the Winsen MH-441D NDIR sensor and the Sensirion SHT40 temperature and relative humidity sensor [26]. PTFE was chosen for its chemical inertness and long-term stability, which prevents alteration of the sample and sensor poisoning. The pneumatic system employs a miniaturised pump driven by a brushless motor in a downstream suction configuration, sized to ensure constant and uniform air exchange. Compared to brushed motors, the brushless solution eliminates the risk of spark generation, representing an essential safety requirement for operation in the presence of high methane concentrations and under hotspot conditions.

All sensors are housed within the same chamber volume and are therefore exposed simultaneously to the same gas sample, rather than sequentially. The sampled air enters through the inlet port, flows across the common sensing volume where the sensors are located, and leaves through the outlet port connected to the miniaturised pump, which operates downstream in a suction configuration so that no active component is located upstream of the sensors. The internal fluid dynamics of the chamber were also tested to assess gas mixing and verify homogeneous exposure of the sensors to the sample.

The control unit is based on an ESP32-WROOM-32 microcontroller, which manages the acquisition of sensor signals at a frequency of 1 Hz via dedicated interfaces: the analogue sensors are read via an external ADS1115 ADC, whilst the digital sensors communicate via I^2^C and UART buses. The printed circuit board (PCB) is a four-layer design, with separation between the analogue and digital sections and a layout optimised to minimise trace lengths and improve immunity to electromagnetic interference. The power management architecture features two independent power paths dedicated to the analogue and digital components, respectively, a solution that significantly reduces electronic noise on analogue sensor measurements and allows the entire system to be powered via a single USB power bank. The acquired data is transmitted to an external device (PC or smartphone) for real-time display and recording. The firmware supports both the standard Bluetooth protocol and Bluetooth Low Energy (BLE), enabling simultaneous communication between multiple ESCAPE Toolbox units and external devices via dedicated software applications. An integrated OLED display also provides real-time information to the operator, including the system status and the overall measured methane concentration.

The entire instrument is housed in a customised casing designed and manufactured using 3D printing, in a compact and robust format, suitable for placement in the bottom compartment of a backpack. The casing consists of two main sections: the upper section contains the circuit board and the pump, whilst the lower section houses the power bank. The result is a fully integrated prototype ready for field use.

**Figure 1:**
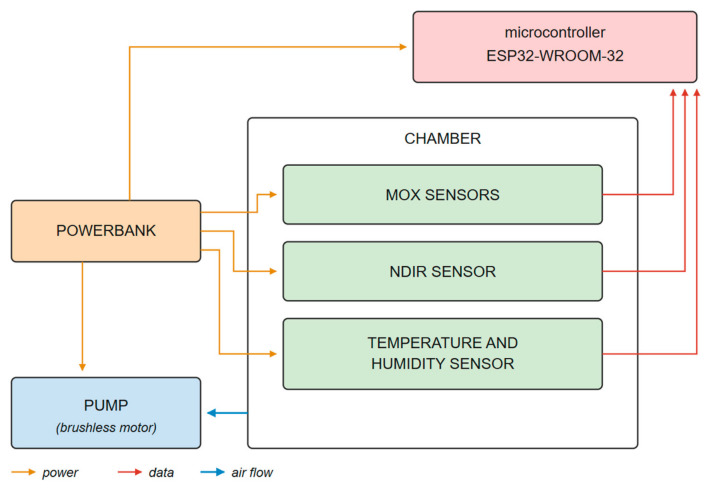
Functional diagram of the Toolbox version V2 and V3.

Figure 1 shows a schematic version of the toolbox.

#### 2.1.3. ESCAPE App

To support data acquisition during walkover surveys, a dedicated mobile application was developed. The application was developed as a native solution for Android operating systems, with the aim of providing field operators with a practical and intuitive tool for managing, viewing in real time and georeferencing the measurements acquired by the toolboxes. The app is based on a client device architecture with direct communication between the smartphone and the toolbox via the Bluetooth Low Energy (BLE) protocol, which ensures continuous data exchange with reduced energy consumption. During use, the app maintains a stable connection with the toolbox’s ESP32 microcontroller, acquiring sensor values at a rate of 1 Hz. Each measurement record is automatically associated with GPS coordinates (latitude, longitude, altitude), a timestamp and the operator’s movement speed and saved in JSON format to the device’s local memory.

The graphical interface is organised into three main screens: a GPS map displaying georeferenced measurement points updated in real time, a screen showing the numerical readings from individual sensors and environmental parameters, and a screen dedicated to time-series graphs of the signals from both measurement chambers. If high concentrations of methane are detected by the MH-441D sensor, the system triggers visual and audible alerts to warn the operator. The application also includes the option to load a pre-trained machine learning model, which processes sensor signals in real time to provide a quantitative estimate of methane concentration directly during the walkover. This feature provides the operator with immediate feedback on the site’s emission conditions, going beyond the simple display of raw sensor signals and making the system usable even by non-specialized personnel. The application also operates in offline mode: in the absence of network connectivity, data is stored locally and automatically synchronised with the ESCAPE cloud platform once the connection is restored, ensuring the continuity of field operations and the structured transfer of data for subsequent analysis.

### 2.2. Laboratory Calibration

#### 2.2.1. Gas Mixing System

Laboratory calibration was carried out using an automated gas mixing system, specifically developed as part of the ESCAPE project to generate gas mixtures with composition and humidity controlled with high precision [27]. The system is equipped with four independent gas lines, each regulated by a dedicated mass flow controller (MFC): a dry air line, used as a dilution gas; a humid air line, obtained by passing air through a bubbler for saturation with water vapour; and two lines dedicated to the target gases, configurable for the introduction of methane and an interfering gas (ethanol, acetone or CO_2_), each supplied from a certified gas cylinder and diluted to the target concentration through the corresponding MFC. The MFCs (Alicat Scientific, Inc., Tucson, AZ, USA) have full-scale ranges of 5 L/min for the two air lines, 2 L/min for the first target-gas line and 0.5 L/min for the second target-gas line. The gas lines are made of Teflon (PTFE) tubing and are not heated. The system operates in fully automated mode: the user sets the desired parameters (target concentrations, relative humidity, total flow rate) via a dedicated spreadsheet, which automatically calculates the required flow rates for each MFC, verifying that the values are compatible with the controllers’ operating limits and optimising the configuration to minimise overall error. Test execution is managed by a Raspberry Pi, which sends setpoints to the controllers and records temperature, humidity, and differential pressure data in real time via reference sensors (SHT40 and SDP810) located immediately upstream of the stilling chamber.

#### 2.2.2. Experimental Setup

**Figure 2:**
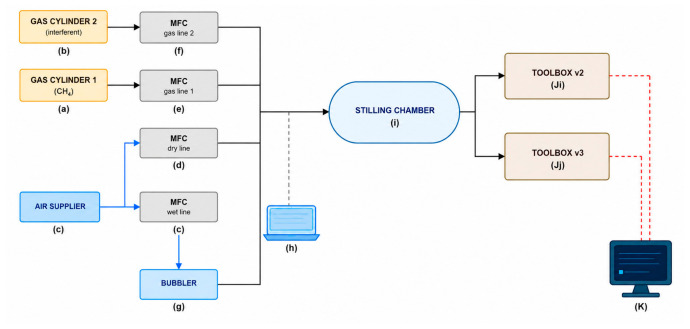
Diagram of the experimental setup used for laboratory calibration, comprising (a) gas cylinder 1, (b) gas cylinder 2, (c) wet line MFC, (d) dry line MFC, (e) gas line 1 MFC, (f) gas line 2 MFC, (g) bubbler, (h) Raspberry and sensors, (i) stilling chamber, (J_i,j_) Toolbox, and (K) computer.

The laboratory calibration tests were conducted simultaneously on the two replicated toolboxes (V2 and V3) so as to expose both units to the same gas mixture and the same environmental conditions. This configuration eliminates the variability associated with separate test sessions and makes the data acquired from the two devices directly comparable. To this end, the stabilisation chamber located downstream of the mixing system was equipped with two independent outlets, each connected to the input of a toolbox. During the tests, the toolbox internal pump was kept active, drawing the gas sample directly from the stilling chamber and each toolbox was powered and controlled by its own ESP32 microcontroller, which acquired sensor signals at a frequency of 1 Hz. An external device was connected simultaneously via Bluetooth to the two toolboxes, enabling real-time visualisation of sensor responses and the collection of two distinct datasets, one for each unit. Figure 2 shows a diagram of the calibration setup.

#### 2.2.3. Test Protocol

The calibration protocol involved two main test types, each designed to characterise a specific aspect of the sensors’ behaviour and following a principle of increasing complexity: first were tests with methane and water vapour only (at varying methane and humidity concentrations) and were second tests with methane and interfering gases other than humidity (at varying concentrations and relative concentrations of the three compounds). Each test was structured as a sequence of constant-concentration steps, each lasting 600 s, which was observed to be a sufficient time to ensure complete stabilisation of the sensor signals. All tests were carried out at a constant total flow rate of 5 L/min, selected to stably supply both toolboxes and ensure rapid renewal of the stilling chamber.

The tests with methane only were carried out by exposing the sensors to controlled concentrations of CH_4_ in synthetic air, in a range from 1 ppm to 1000 ppm. Each test consisted of 18 steps and comprised two phases: an initial phase of progressive step-up through seven concentration levels (1, 5, 10, 50, 100, 500 and 1000 ppm), followed by a phase of absolute steps in which each level is applied individually with a return to the baseline (1 ppm) between one step and the next. The tests were conducted at four relative humidity levels (20%, 40%, 60% and 80% RH), with two replicates for each level, for a total of eight tests, as shown in Table 2.

Tests with interfering gases were conducted to assess the cross-sensitivity of MOX sensors to compounds that could potentially be present in landfill emissions. The choice of interferents was guided by some considerations about the composition of landfill gas (LFG), which typically is more than 90% CH_4_ and CO_2_ in approximately equal proportions, with trace amounts of VOCs [1]. CO_2_ therefore represents the main potential interferent, being present at concentrations comparable to methane. Among VOCs, ethanol and acetone have been considered because, besides being frequently detected in landfill surface emissions [28], they are known to elicit particularly strong responses in MOX sensors even at low concentrations [18], making them critical interferents for assessing the system’s selectivity. The tests followed a standard protocol based on a concentration matrix comprising 31 steps of 10 min each, for a total duration of approximately 5 h per test. For each methane level (10, 50, 100, 500 and 1000 ppm), three steps with increasing concentrations of the interferent were applied, with interferent/methane ratios representative of the conditions found near the landfill surface. Clean air (baseline) steps and steps with only the interferent (without methane) were inserted between each group to isolate the specific contribution of each compound to the sensor response. Each test set was conducted at two relative humidity levels (30% and 60% RH).

Table 3 and Table 4 show the calibration protocols of the tests with methane, the tests with acetone, and the tests with carbon dioxide.

Table 5 summarises the entire campaign.

### 2.3. Field Measurement Campaigns

The sensor toolboxes developed as part of the project were further trained and validated through field measurement campaigns carried out at two different active Italian landfills, with the dual objective of verifying their behaviour under real operating conditions and collecting the datasets necessary for the development, calibration, validation and testing of machine learning models (as described in Section 2.4).

**Figure 3:**
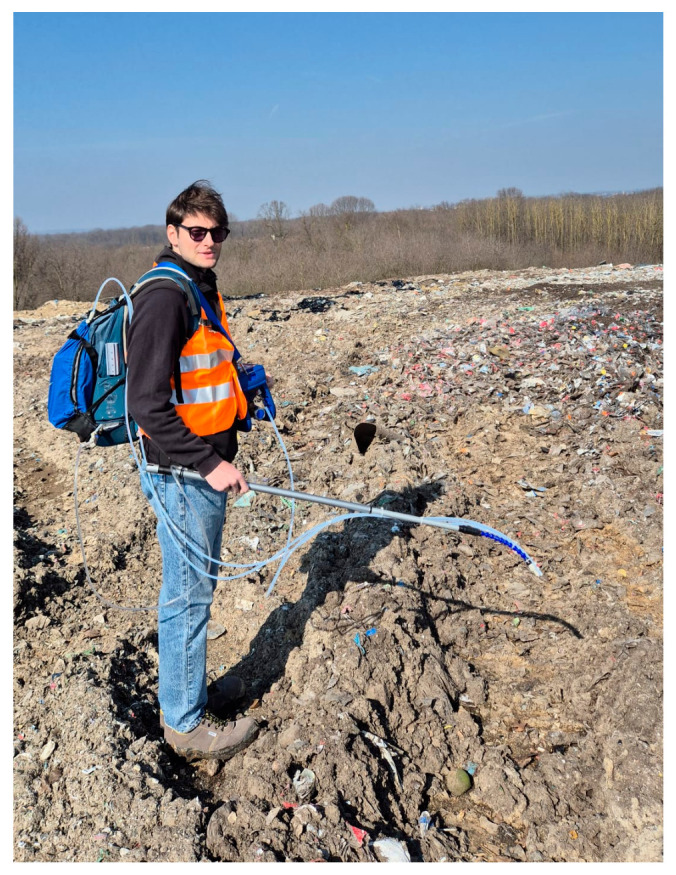
Portable setup adopted during field walkovers.

To enable the use of the toolboxes during mobile monitoring activities, a dedicated portable sampling system was developed, designed to ensure portability, ease of use and reliability of sampling by a single operator. The system consists of two main components: a backpack, which serves as a support for transporting the equipment, and a telescopic probe, used to collect the gas sample near the surface of the landfill. As shown in Figure 3, the two components can be seen worn and used in the field.

Both sensor toolboxes (V2 and V3) were housed inside the backpack, arranged in the two internal compartments designed to hold the units in a stable and protected position. The telescopic probe consists of an extendable rod housing a flexible Tygon tube; this material was chosen for its chemical inertness and suitability for transporting the gas sample without significantly altering its composition. The lower end of the tube is connected to the toolbox inlets, whilst the upper end of the probe constitutes the sampling point, which the operator positions manually a few centimetres from the surface of the landfill site, following the walkover routes. The ability to extend and retract the rod allows the operator to adjust the sampling height to the terrain and to maintain an ergonomic posture throughout the survey.

To enable simultaneous comparison between the two toolboxes and a reference instrument, together with the two toolboxes we used a portable flame ionisation detector (FID) (Gastec, Crowcon Detection Instruments Ltd., Abingdon, UK) [29]. One main drawback associated with the use of a portable FID as a reference instrument, is that this instrument is not selective to methane, meaning that the FID’s readings in ppm will result from both methane and any other compound undergoing combustion in an H_2_ flame. Despite this well-known limitation, the use of the FID was deemed reasonable, because this is currently the “gold-standard” device for walkover surveys [30]: indeed, the only other compound that is present at comparable concentrations to methane in landfill gas is CO_2_, which is not measured by the FID. The inlet tubes of both toolboxes and the FID’s suction tube were connected at the top of the telescopic pole, causing all the tubes to converge at the same sampling point. This unified sampling configuration ensures that the three devices simultaneously analyse the same air mass, making the recorded signals directly comparable and limiting the influence of spatial variability. During monitoring activities, the operator manually held the telescopic probe, positioning the sampling point a few centimetres from the surface of the landfill and following the walkover routes in the various areas of the sites.

Data were collected by means of the ESCAPE mobile app, with each toolbox connected via Bluetooth to a dedicated smartphone. Each unit recorded sensor signals in real time, at a frequency of 1 Hz, along with GPS coordinates, timestamps and the operator’s movement speed. In parallel, the FID readings were recorded by its internal data logger. During post-processing, the datasets produced by the toolboxes and those from the FID were synchronised in time, allowing a direct comparison between the signals from the low-cost sensors and the FID measurements, considered as the reference. This approach enabled the construction of a robust database for subsequent comparative analyses and for training predictive models.

The measurement campaigns were conducted at two active Italian landfills, selected to verify the robustness of the system under different environmental, meteorological and operational conditions, and to enrich the training dataset with the widest possible variety of scenarios. Three walkovers were carried out at Landfill A; the first two, designated NW1 and NW2, were conducted on the same day, in the morning and afternoon, respectively, under stable but progressively warmer conditions. The routes covered are characterised by different types of surface cover, including zones with temporary cover, active disposal areas and sections with permanent cover. The third walkover, NW3, was carried out on a different day at the same site, under significantly different weather conditions compared to NW1 and NW2, with the aim of verifying the repeatability of the measurements and observing the system’s behaviour as ambient temperature and humidity varied.

Finally, walkover NW4 was carried out at a different landfill (Landfill B), divided into two distinct sessions, NW4_1 and NW4_2. The inclusion of a second experimental site was deemed essential to assess the generalisability of both the measurement system and the calibration models, verifying their applicability even in contexts different from those used in the initial development phase. Landfill B also exhibits emission characteristics distinct from those of Landfill A: during the walkovers carried out at this site, the sensors and FID more often detected emission hotspots with higher methane concentration peaks than those observed at Landfill A, providing the dataset with measurements at the upper extremes of the concentration range explored in the field. This combination of different weather conditions and a wider range of methane concentrations played a key role in improving the calibration model, as it exposed the toolboxes to a more diverse set of operating conditions, reducing the risk of overfitting to the conditions of a single site.

### 2.4. Machine Learning Model for Estimating CH_4_ Concentration

A regression model was developed to estimate methane (CH_4_) concentration directly from the raw sensor signals. The method chosen is XGBoost (eXtreme Gradient Boosting), an approach based on decision trees with gradient boosting that sequentially constructs an ensemble of numerous shallow trees, in which each new tree is trained to reduce the residual errors made by the previous ones.

The concentration was estimated using the raw signals from the methane-sensitive sensors installed in each toolbox as input features to the XGBoost regressor, while the FID-measured concentration was used as the target value. The sensor signals were not averaged or manually combined before modelling; instead, their combined response was learned directly by the algorithm, which maps the multivariate sensor signal pattern to a single predicted concentration.

More formally, the model’s final prediction is obtained as the sum of the outputs of the individual trees:

(1) $${\hat{y}}_{i}=\sum_{k=1}^{K}f_{k}(x_{i}),f_{k}\in F$$

where $x_{i}$ is the vector of input features (the sensor signals), K is the total number of trees in the ensemble, and F is the regression tree space. Training is carried out by minimising an objective function that combines a loss term (mean squared error, in this regression context) and a regularisation term that penalises the complexity of the trees, helping to reduce the risk of overfitting [31].

This strategy is particularly well suited to sensor datasets as it allows for the capture of non-linear relationships and interactions between features without requiring strict assumptions about the data distribution, whilst maintaining good robustness on tabular data. These properties are particularly relevant in the application context under consideration, given the well-known non-linearity of the MOX sensors’ response to the concentration of the target gas and the presence of interactions with environmental variables such as relative humidity.

The XGBoost regressor was configured with the following hyperparameters. The number of boosting rounds was set to 800 (n_estimators = 800), i.e., the number of regression trees built sequentially, each trained to correct the residual error of the previous ones. The maximum depth of each tree was limited to 8 (max_depth = 8), a value that controls the model complexity and the order of the feature interactions a single tree can represent.

The learning rate was set to 0.05 (learning_rate = 0.05), a shrinkage factor that reduces the weight of each tree to achieve a more gradual and stable learning, at the cost of a larger number of trees. To limit overfitting, each tree was grown on a random 80% subsample of the training instances (subsample = 0.8), while all input features were retained at every split (colsample_bytree = 1.0), given their limited number. The leaf weights were regularised with an L2 penalty (reg_lambda = 1.0), which discourages large weights and improves generalisation, whereas the L1 penalty was disabled (reg_alpha = 0). Finally, the histogram-based tree construction algorithm (tree_method = “hist”) was adopted, which discretises the continuous feature values into bins to speed up training with a negligible impact on accuracy.

The model’s performance was assessed through repeated train/test evaluations across different acquisition sessions (‘walkovers’) and the two toolboxes (V2 and V3), in order to maximise the variability of operating conditions and quantify the uncertainty associated with the estimates. This evaluation scheme was specifically designed to analyse two sources of variability critical to the target application: inter-session variability, which is associated with differing environmental, meteorological and operational conditions between walkovers and sites, and inter-device variability, which is associated with manufacturing tolerances and minor construction differences between nominally identical units. Testing the model on walkovers and toolboxes excluded from the training set therefore provides a direct assessment of the calibration’s transferability, a fundamental requirement for the mass production of multiple units without the need for individual calibration.

In the presence of missing values in the sensor signals, a simple and robust pre-processing step was adopted based on median imputation, calculated on the dataset used for training/testing in each run.

The main evaluation metrics considered were $R^{2}$, MAE and RMSE, defined as follows:

Coefficient of determination ($R^{2}$):

(2) $$R^{2}=1-\frac{\sum_{i=1}^{n}(y_{i}-{\hat{y}}_{i}{)}^{2}}{\sum_{i=1}^{n}(y_{i}-\overset{ˉ}{y}{)}^{2}}$$

where $y_{i}$ and ${\hat{y}}_{i}$ are, respectively, the reference concentration (measured by the FID) and the concentration estimated by the model for the i-th sample; $\overset{ˉ}{y}$ is the mean of the reference values; and n is the number of samples in the test set. The $R^{2}$ quantifies the proportion of the target variable’s variance explained by the model, with $R^{2}=1$ indicating a perfect prediction.

Mean Absolute Error (MAE):

(3) $$\mathrm{MAE}=\frac{1}{n}\sum_{i=1}^{n}|y_{i}-{\hat{y}}_{i}|$$

The MAE represents the average error made by the model, expressed in the same units as the target variable (ppm), and weights all errors linearly, making it robust to outliers.

Root Mean Square Error (RMSE):

(4) $$\mathrm{RMSE}=\sqrt{\frac{1}{n}\sum_{i=1}^{n}(y_{i}-{\hat{y}}_{i}{)}^{2}}$$

The RMSE, also expressed in ppm, penalises large errors more heavily than the MAE, due to its quadratic nature. Comparing the MAE and RMSE provides information on the distribution of errors: similar values indicate errors of similar magnitude, whilst an RMSE significantly greater than the MAE indicates the presence of large errors, typically associated with concentration peaks at emission hotspots.

In addition to these scalar metrics, a time-series analysis was also carried out by comparing the values predicted by the model with the FID reference measurements throughout the entire duration of each walkover. This analysis allows for the evaluation of aspects not captured by the aggregate metrics, such as the model’s ability to correctly identify the temporal position (and thus, given the georeferencing of the data, the spatial position) of emission hotspots, the correct reconstruction of the shape of concentration peaks, and the possible presence of systematic biases at low concentrations.

## 3. Results and Discussion

This section presents the results of the predictive analysis and the transferability of the calibration model between the two replicated units of the sensor toolbox, as well as the effect of including laboratory data in the training set.

The aim is to verify the two hypotheses underlying the scale-up strategy: (i) that the developed model is able to predict FID concentration (C__FID_) with satisfactory accuracy to make the identification of emission hotspots over a landfill surface, and (ii) that units built with the same specifications and trained with the proposed lab + field approach exhibit sufficient reproducibility to allow the prediction model to be transferred from one device to the other.

To isolate these components, the analysis was set up using a train/test protocol in which the NW3 walkover was used as the test set, whilst the NW1, NW2, NW4_1 and NW4_2 walkovers were used for training. The choice of NW3 as the hold-out walkover is motivated by its particularly balanced concentration distribution: NW1 and NW2 are dominated by relatively low methane concentrations, representative of emissions from roof surfaces, whilst NW4_1 and NW4_2, conducted near identified hotspots, provided the high-concentration events necessary to expose the model to the upper part of its operational range. All combinations of training and test toolboxes within the V2/V3 family were considered, for a total of four train → test pairs, and the analysis was replicated in two configurations: a first scenario in which the training set comprises exclusively field data, and a second scenario in which laboratory calibration data acquired on the same toolbox used for training are added to the field data. This was carried out with the specific purpose of verifying that the lab calibration based on the acquisition of highly reproducible data under controlled conditions using the dedicated gas mixing system is useful in improving predictive performance in both scenarios.

As a final step, we carried out a preliminary analysis to evaluate the “delay” between the FID responses and the toolbox predictions, with the purpose of acquiring some preliminary observations to understand how much of the residual discrepancy between the two concentration values (predicted vs. “true” value measured by the FID) could potentially be attributed to a different response dynamics of the two devices, knowing that MOX sensors have a slower response compared to the FID.

The model performances were evaluated both by comparing the predicted time series with the FID measurements and using aggregate regression metrics (R^2^, MAE and RMSE).

### 3.1. Sensor Response to Laboratory Calibration

Before assessing the predictive model, the raw response of the sensors was examined under the controlled laboratory conditions described in Section 2.2 in order to verify that the selected sensors respond to methane across the target range and to characterise their behaviour in the presence of interferents.

**Figure 4:**
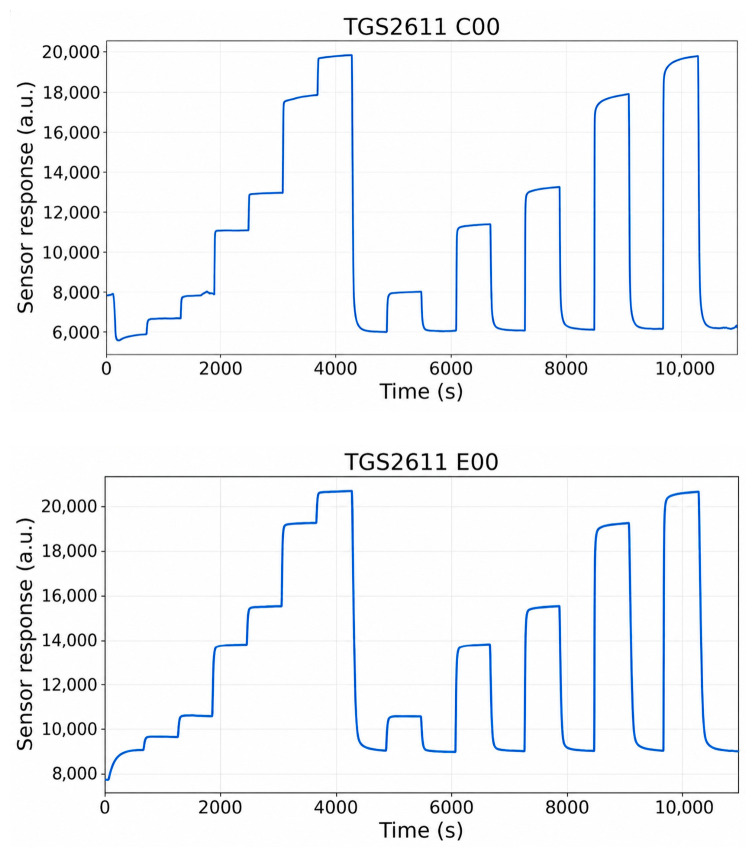
Raw response of the TGS2611 sensors (C-00 and E-00) of toolbox V2 during the methane step protocol, carried out at 40% relative humidity (Table 2).

During the methane-only protocol (Table 2), both TGS2611 variants reproduced the concentration steps with fast response and recovery and a stable baseline between steps, with a clearly detectable signal already at the lowest steps (down to the order of 5 ppm). The C-00 variant showed the largest response, while the filtered E-00 gave a slightly lower but very consistent signal. The MH-441D NDIR sensor, consistent with its higher detection threshold, responded appreciably only to the highest steps (≈1000 ppm), confirming its role as a high-concentration reference rather than a low-ppm detector. Figure 4 shows the response curves for toolbox V2; toolbox V3 exhibited entirely analogous behaviour, as reported in the Supplementary Materials (Figures S3–S5), confirming the reproducibility on which the calibration transfer strategy relies.

**Figure 5:**
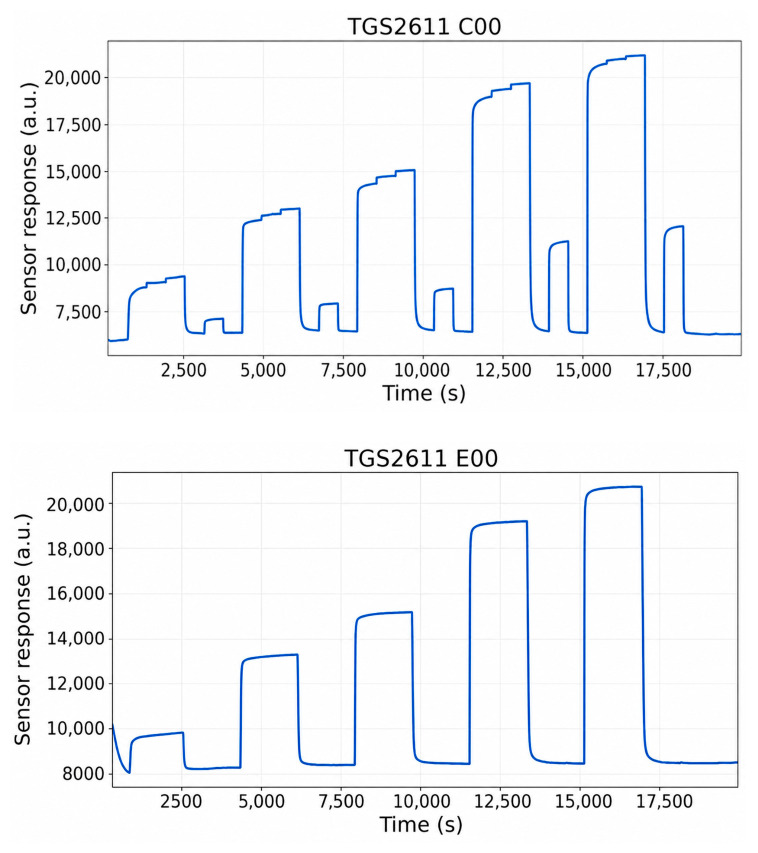
Response of the TGS2611 sensors (C-00 and E-00) to the acetone interference test, carried out at 30% relative humidity (Table 3).

The sensor response was then characterised in the presence of interferents, to evaluate the residual cross-sensitivity of the MOX sensors. Figure 5 reports an example based on the acetone test (Table 3), where the two variants show markedly different behaviour: the C-00, lacking the filter, detects the acetone and produces an appreciable response, whereas the E-00, thanks to its activated carbon filter, does not respond to the interferent. This difference between the two channels provides information that the machine-learning model can exploit to distinguish methane from interferents and mitigate cross-sensitivity.

The complete set of response curves for both toolboxes covering the methane-only tests, the tests with all the considered interferents (CO_2_, ethanol and acetone), and the temperature and relative humidity recorded inside the measurement chamber are reported in the Supplementary Materials (Figures S1–S11).

### 3.2. Model Prediction Accuracy for the Two Devices

**Figure 6:**
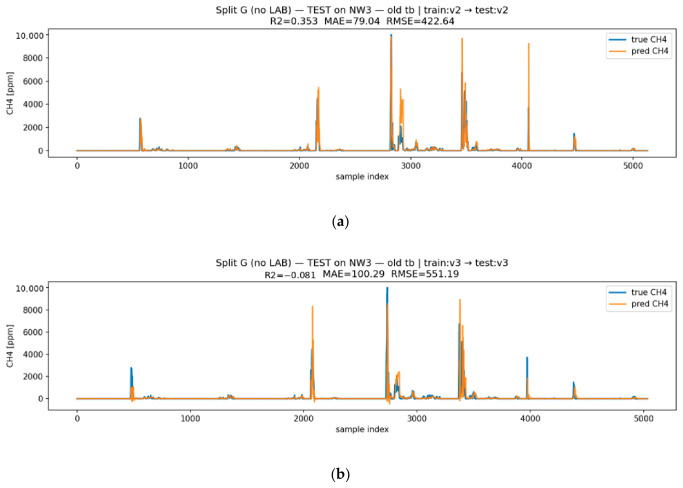
Comparison between concentration measured by the FID (blue line) and predicted by the model trained on field data (orange line) on the NW3 walkover: (**a**) train V2 → test V2, (**b**) train V3 → test V3. In the plots, “true CH_4_” indicates the concentration measured by the FID, while “pred CH_4_” indicates the corresponding concentration predicted by the model.

In this first phase, the performance of the model developed for the quantitative estimation of C__FID_ on the two toolboxes V2 and V3 is evaluated. The training set consists of walkovers NW1, NW2, NW4_1 and NW4_2, whilst walkover NW3 is used as an independent test set (Figure 6). The time series predicted by the model are compared with the FID measurements acquired simultaneously during the field campaign.

The results obtained for the two toolboxes are shown in Figure 6. For both units, the model reproduces the temporal trend of the FID signal throughout the entire duration of the walkover, correctly identifying both the low-concentration baseline sections and the peaks associated with passing near hotspots. The amplitudes of the predicted peaks are consistent with those measured by the FID, even for the most “intense” hotspots. In field conditions, the minimum detectable C_FID was approximately in the order of 10 ppm (up to ~20 ppm depending on environmental conditions), compared with the ~5 ppm limit obtained in laboratory tests. This result further supports the applicability of MOX sensors for real-world methane hotspot detection.

The aggregate metrics obtained on the test set indicate an R^2^ of 0.35, an MAE of 79.0 ppm and an RMSE of 422.6 ppm for the V2 toolbox and an R^2^ of −0.08, an MAE of 100.3 ppm and an RMSE of 551.2 ppm for the V3 toolbox. The MAE values, in the tens of ppm range, confirm that the typical error of the model on individual samples is limited and consistent with good tracking of concentration dynamics throughout most of the walkover.

The marked discrepancy between MAE and RMSE for both units indicates that the overall error is not uniformly distributed but is dominated by a limited number of high-deviation events, associated with isolated overestimates at high-concentration peaks. To characterise this, the test set error was examined separately across three concentration ranges (0–50, 50–200 and >200 ppm; Table 6), the low range being the most relevant for hotspot detection. The error remains small in the 0–50 ppm range, which contains most of the samples, and grows markedly above 200 ppm, where it weighs disproportionately on RMSE and R^2^. This high range is, however, strongly under-represented (~6% of the samples), as walkovers cross hotspots only briefly, which limits the information available to the model in that regime. Table 6 shows the metrics in their respective ranges.

The origin of part of this residual error is examined in Section 3.2.2, in relation to the different response dynamics of the sensors and the FID.

#### 3.2.1. Effect of Laboratory Calibration on Model Performance

The analysis continued by integrating into the training set the calibration data acquired in the laboratory on the same toolbox, with the aim of verifying whether the addition of such data leads to some improvement in the model’s predictive performance compared to the training based solely on field data. The test set configuration was kept unchanged, using the NW3 walkover as the hold-out, to make the metrics obtained in the two scenarios directly comparable.

The laboratory subset added to the training set included both the methane-only tests and the interferent tests (CO_2_, ethanol and acetone), covering the full lab calibration campaign.

**Figure 7:**
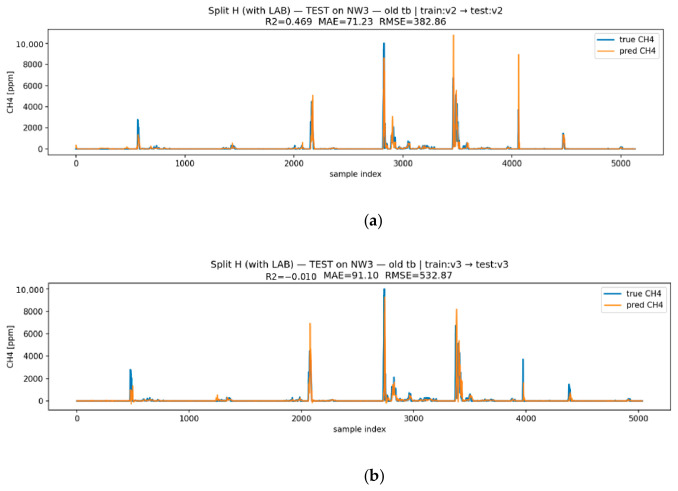
Comparison between concentration measured by the FID (blue line) and predicted by the model trained on field and laboratory data (orange line) on the NW3 walkover: (**a**) train V2 → test V2, (**b**) train V3 → test V3. In the plots, “true CH_4_” indicates the concentration measured by the FID, while “pred CH_4_” indicates the corresponding concentration predicted by the model.

The results obtained by adding the laboratory data to the training set are shown in Figure 7. For both toolboxes, the qualitative tracking of the FID signal is improved compared to the case without laboratory data, as the baseline appears more stable in low-concentration regions, and peak events are reproduced with greater adherence to the FID measurements, even in the high-concentration range.

In quantitative terms, for the V2 toolbox, there is an increase in R^2^ from 0.35 to 0.47, a reduction in MAE from 79.0 to 71.2 ppm, and a reduction in RMSE from 422.6 to 382.9 ppm. For the V3 toolbox, R^2^ changes from −0.08 to −0.01, MAE from 100.3 to 91.1 ppm, and RMSE from 551.2 to 532.9 ppm.

The improvement in metrics, observed consistently for both units, confirms that the inclusion of laboratory data in the training set contributes positively by increasing the model’s predictive capability.

This improvement could be attributed to the fact that the laboratory data extend the operational domain covered by the training set, since the conditions encountered during the walkovers, whilst representative of the real-world deployment scenario, cover only a limited portion of the sensor’s operational space. In particular, the relative humidity levels recorded during the field campaigns were determined by the prevailing weather conditions and did not allow the model to be exposed to controlled variability of this parameter. The laboratory tests, comprising measurements at controlled concentrations of methane in a range between 1 and 1000 ppm at relative humidity levels varying between 20% and 80% provide the model with additional information that allows the C__FID_ to be predicted with better accuracy.

The extent of the improvement, whilst significant, is not, however, sufficient to substantially alter the overall picture: the R^2^ value for the V3 toolbox remains close to zero, suggesting that a significant portion of the residual error is not attributable to the coverage of the operating domain but to another systematic source, which will be analysed in the following section.

#### 3.2.2. Preliminary Analysis of the Time Delay Between the Predicted Concentrations and the FID Measurements

**Figure 8:**
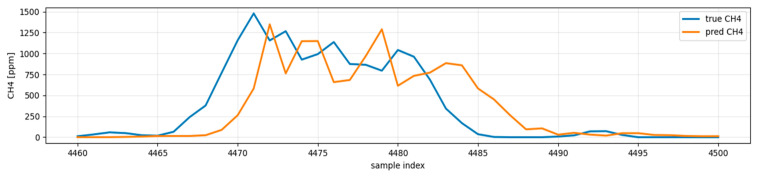
Detail of a peak event from the NW3 walkover, highlighting the time delay of the predicted signal (orange line) with respect to the FID reference (blue line). In the plots, “true CH_4_” indicates the concentration measured by the FID, while “pred CH_4_” indicates the corresponding concentration predicted by the model.

In order to better understand the reasons for such relatively low values of the aggregate regression metrics (especially the coefficient of determination R^2^) despite a very good capability of the predicted concentrations to “follow” the trend measured by the FID and identify the same hotspots; as is visible from the comparison of the time series illustrated in Figure 6 and Figure 7, we tried to further analyse our results by “zooming” into the predictions related to the different peaks detected. By changing the time scale and looking closer to each peak, we could observe, for both toolboxes, the existence of a small systematic time delay between the concentrations predicted by the model and those measured by the FID. This delay is evident both at the rising and falling edges of the concentration peaks, where the predicted signal tends to lag behind that of the FID by a few seconds. Figure 8 shows a detail of the time series corresponding to one of the peak events observed during the NW3 walkover.

The concentration predicted by the toolbox follows that of the FID with a systematic delay of a few seconds. This delay is most likely related to the different response dynamics of the two measurement systems: the FID response to the presence of combustible gaseous compounds is indeed significantly faster than that of MOX sensors responses.

Based on this observation, a preliminary and “rough” parametric analysis was conducted with the aim of quantifying the impact of this delay on the aggregate metrics and thus providing some insight for distinguishing the error component attributable to this time lag from the model’s intrinsic predictive component. To do this, in a very simplistic manner, the predicted concentration was time-shifted relative to the FID measurement by a variable number of samples (from −6 to +3 s, considering an acquisition frequency of 1 Hz), and the regression metrics were recalculated for each shift value. The analysis was conducted using the model trained with the training set including also the laboratory data, as described in the previous section.

**Figure 9:**
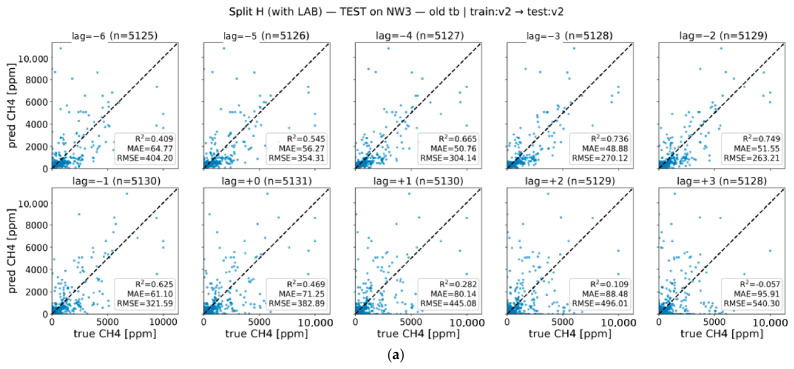

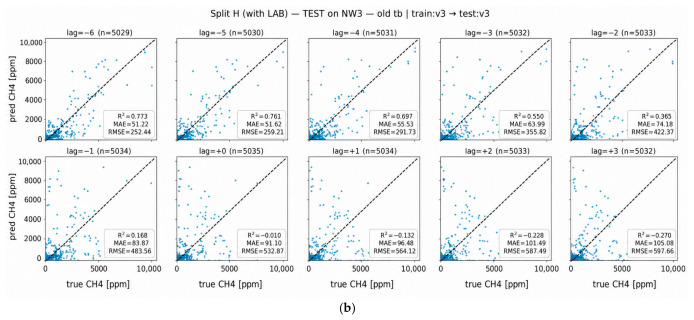
Scatter plots comparing the CH_4_ concentration values measured by the FID (“true”) and those predicted by the model (“pred”) considering the NW3 walkover as a test set, reported for different values of the time shift value applied to the predicted concentration: (**a**) train V2 → test V2, (**b**) train V3 → test V3. The dashed line indicates the bisector (y = x).

Figure 9 shows the scatter plots comparing predicted and “true” concentrations (i.e., those measured by the FID) for the different shift values tested and for both toolboxes. The analysis shows that applying a negative shift, which means “anticipating” the predicted concentration with respect to the FID measurements, results in a progressive narrowing of the scatter plot around the bisector, with an improvement in the metrics, corresponding to an increase in the value of the coefficient of determination R^2^) up to an optimal value, beyond which performance decreases again. Table 7 reports the comparison between the applied lag and the model metrics for the V2 → V2 configuration, while Table 8 reports the same comparison for the V3 → V3 configuration; in both cases, the model was trained by including the laboratory calibration data.

From the data reported in Table 7 and Table 8, it is possible to observe that this very rough lag correction produces a marked improvement in all aggregate metric for both toolboxes, with an optimal performance achieved with a shift of −2 s for toolbox V2, and −6 s for toolbox V3. It is also worthy highlighting that the values of the performance indexes achieved for both toolboxes at the optimal shift values are not only very satisfactory but also very comparable to each other: for both toolboxes, the model achieves an R^2^ between 0.75 and 0.77, an MAE of approximately 51 ppm, and an RMSE of around 250–260 ppm. These values confirm the model’s good predictive capability; moreover, the consistency of the R^2^, MAE, and RMSE values obtained for the two units also indicates that the intrinsic performance of the model is fully comparable between V2 and V3, confirming the reproducibility of the response between units built to the same specifications, as will be further discussed in the next section.

These results indicate that a very significant proportion of the error observed at zero shift is not attributable to an actual limitation of the model in estimating C__FID_ but is the result of a “delay” between the two predictions, which is likely related to the different response dynamics of the two measurement systems. Indeed, the response of the FID to the presence of combustible gaseous compounds is almost instantaneous, while the response of MOX sensors is typically in the order of magnitude of 1 min [32]. The different response dynamics of the two measurement systems is clearly partially accounted for in the model training, since FID measurements are used as the reference for concentration when training the model with field data (which is the reason why the “delay” observed in this study is of a few seconds and not of one minute); however, the different methane adsorption and desorption dynamics on the sensor surface as well as the high amount of “zeroes” registered in the field campaigns may have contributed to such observed delays remaining between the two measurements. These observations lead to the need for a deeper investigation of these aspects in the future, but this was considered out of the scope of this work, which represents a preliminary functionality evaluation of the system developed.

### 3.3. Model Transferability Between the Two Toolboxes

The previous sections show that the model, once trained and tested on data collected by the same toolbox, can estimate C__FID_ with good accuracy. As a further step, this section provides a preliminary evaluation of the model’s ability to maintain comparable performance when applied to a toolbox different from the one used for training, which is a scenario of high practical interest. In view of the production and deployment of multiple units, avoiding individual calibration for each new device is a fundamental requirement for system scalability.

**Figure 10:**
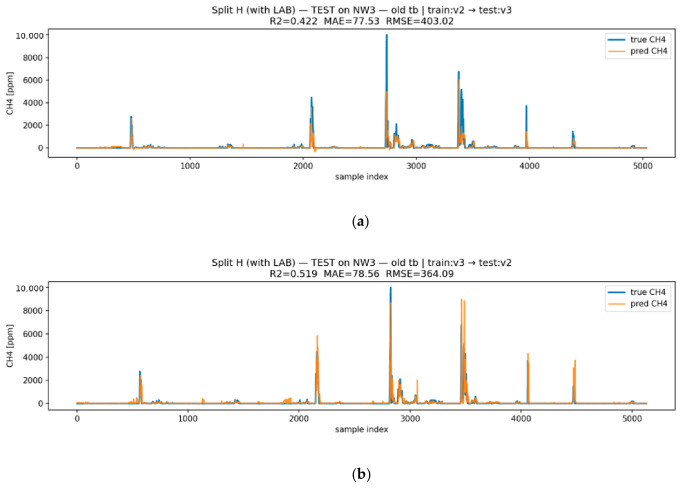
Comparison between concentration measured by the FID (blue line) and predicted by the model (orange line) on the NW3 walkover: (**a**) train V3 → test V2, (**b**) train V2 → test V3. In the plots, “true CH_4_” indicates the concentration measured by the FID, while “pred CH_4_” indicates the corresponding concentration predicted by the model.

The analysis was conducted by considering the two complementary cross-device configurations for testing: the model trained on data collected by toolbox V2 was tested on the data collected by toolbox V3 (V2 → V3), and the model trained on V3 was tested on V2 (V3 → V2), whilst keeping the train/test protocol described in the previous sections unchanged, with the NW3 walkover used as the hold-out for testing and the laboratory data included in the training set. Figure 10 shows the time series obtained in the two cross-device tests.

**Figure 11:**
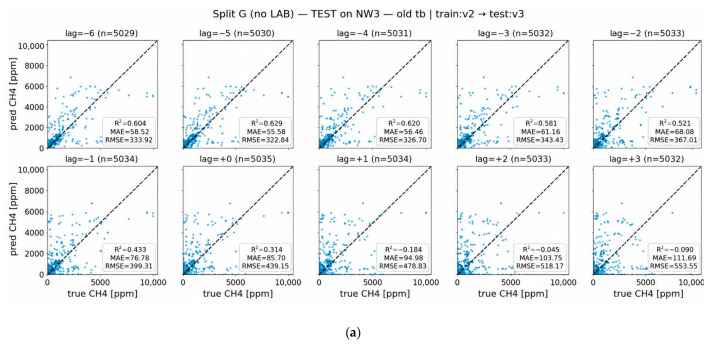

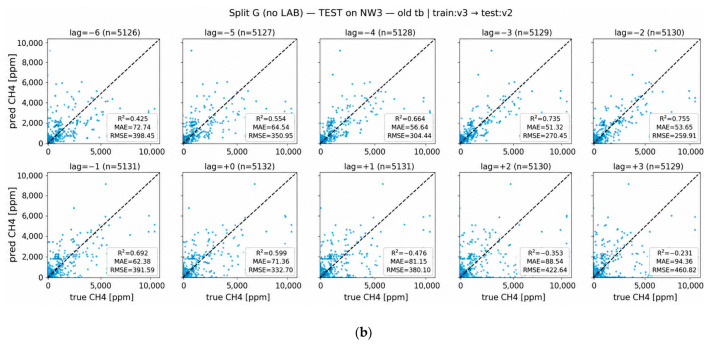
Scatter plots comparing the concentration values measured by the FID (“true”) and those predicted by the model (“pred”) considering the NW3 walkover as a test set, as reported for different values of the time shift value applied to the predicted concentration: (**a**) train V3 → test V2, (**b**) train V2 → test V3. The dashed line indicates the bisector (y = x).

In both cases, the model correctly identifies the sequence of emission events along the walkover and reproduces their amplitudes in good agreement with the FID measurement, demonstrating that predictions developed on one device retain their validity even when applied to a different unit. The V3 → V2 configuration shows particularly accurate tracking, with good adherence to the reference both in baseline sections and at peaks. The V2 → V3 configuration, on the other hand, shows a tendency to underestimate high-concentration peaks, whilst the tracking of medium and low-intensity peaks and baseline sections remains consistent with the reference. In quantitative terms, the V3 → V2 configuration achieves an R^2^ of 0.52, an MAE of 78.6 ppm and an RMSE of 364.1 ppm, whilst the V2 → V3 configuration achieves an R^2^ of 0.42, an MAE of 77.5 ppm and an RMSE of 403.0 ppm; these values confirm the model’s ability to correctly predict C__FID_ even in a cross-device scenario, albeit with a margin for improvement that has been examined through analogous considerations about the delay between FID measurements and model predictions previously described in Section 3.2.2. A similar analysis was performed by artificially introducing a time shift ranging from −6 to +3 s and is presented in Figure 11.

The observed trend is consistent with that described in the previous section for same-device configurations: the application of a negative shift produces a progressive contraction of the scatter plot around the bisector. In this case, for the V3 → V2 configuration, the optimal shift value is −2, increasing R^2^ from 0.52 to 0.77, MAE from 78.6 to 59.6 ppm and RMSE from 364.1 to 253.4 ppm, while for the V2 → V3 configuration, the optimal shift value is −2 samples, increasing R^2^ from 0.42 to 0.49, MAE from 77.5 to 63.8 ppm and RMSE from 403.0 to 379.0 ppm.

**Figure 12:**
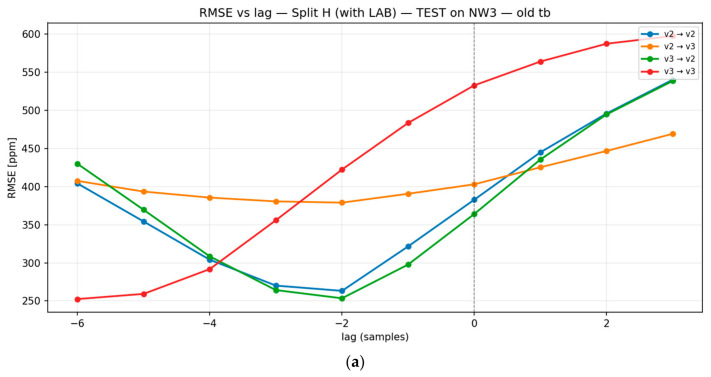

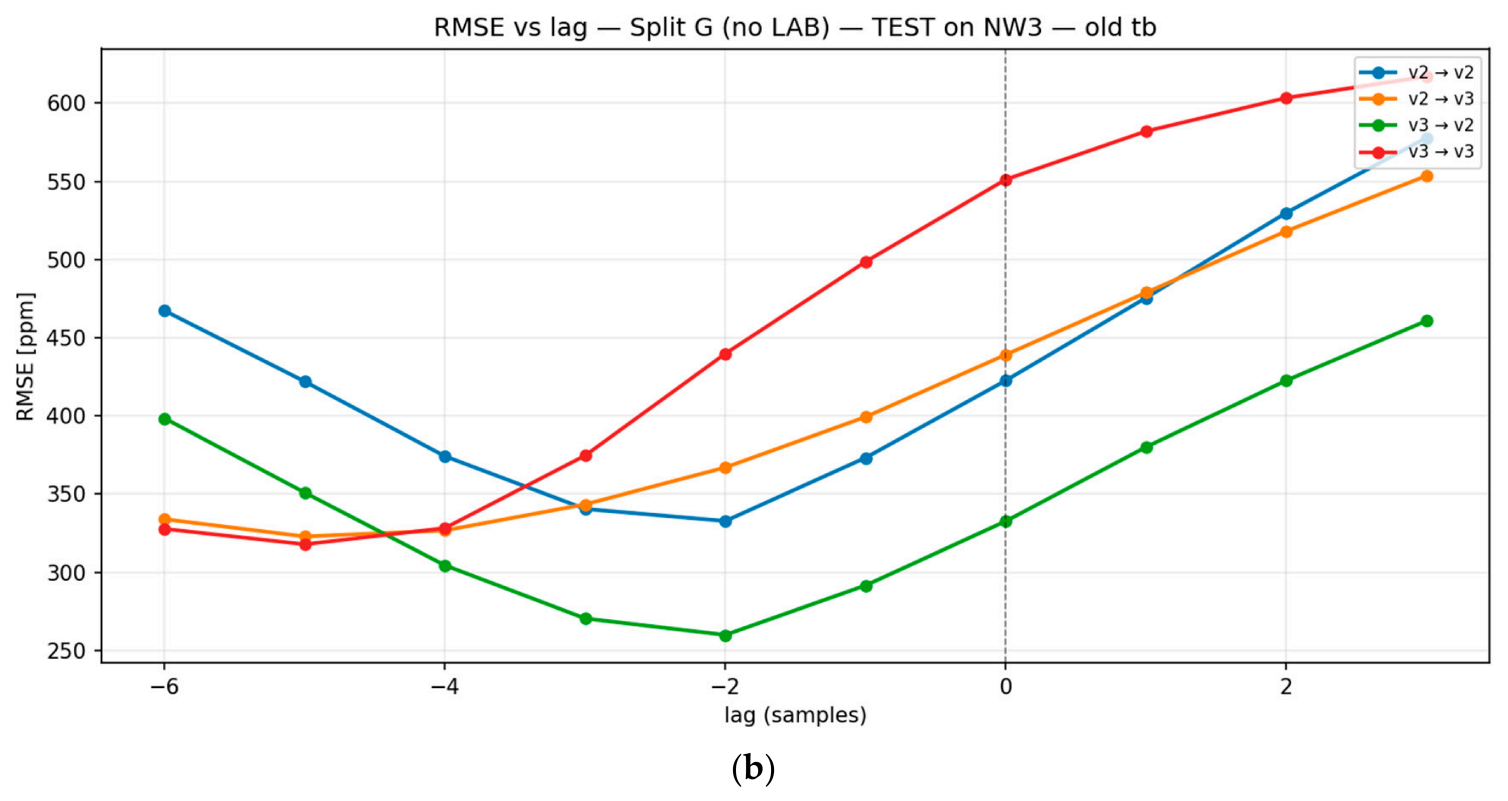
RMSE as a function of the time shift values applied to the predicted concentrations for the four train–test combinations analysed: (**a**) model trained on field data only, (**b**) model trained on field and laboratory data.

As a further analysis, we compared the optimal shift values derived for the four train → test combinations (V2 → V2, V3 → V3, V3 → V2, V2 → V3) in both training scenarios considered in this work: the one based solely on field data and the one that also includes laboratory calibration data. The comparison is made by plotting the RMSE value (in ppm) as a function of the different time shift values considered (Figure 12).

From the plots shown in Figure 12, it can be seen that the optimal shift value is determined solely by the test toolbox and is independent of the device used for training: when the test is performed on the V2 toolbox, the optimal lag is −2 samples in both the V2 → V2 and V3 → V2 configurations, whilst when the test is performed on the V3 toolbox, the optimal lag lies between −5 and −6 samples in both the V3 → V3 and V2 → V3 configurations. This observation seems to indicate that the delay may be related to some physical characteristic of the individual hardware unit. Although the two toolboxes were built to the same specifications (same sensors, same measurement chamber, same firmware, same sampling pump and same sampling line), there may still be some differences in overall response times attributable to manufacturing tolerances on the mechanical components, minor variations in the effective length of the sampling line, and the inherent variability in the response of the individual MOX sensors.

It is important to highlight that, after applying a suitable time shift as described above, the model trained on a toolbox is capable of delivering performance comparable to that obtained in a same-device configuration, as demonstrated by the V3 → V2 case, where R^2^ reaches a value of 0.77, which is substantially identical to that of the V2 → V2 case.

It should be noted that the optimal shift is identified only through comparison with the reference itself, and its direct application would therefore require knowledge of future values of the reference signal. The time-shift analysis is thus to be regarded as a preliminary diagnostic carried out within the exploratory data-processing stage of this study. Rather than representing a real-time correction, its main value is interpretative, since the marked improvement in the metrics obtained at the optimal shift indicates that a substantial part of the residual discrepancy between predicted and measured concentrations stems from the different response dynamics of the two measurement systems rather than from an intrinsic limitation of the model, which therefore retains a genuine underlying predictive capability. Building on this preliminary evidence, future developments will address the phenomenon in a more rigorous way, embedding the response dynamics directly in the model through, for instance, the inclusion of dynamic features (moving averages or signal derivatives), the adoption of recurrent architectures (LSTM or GRU networks), or the explicit characterisation and compensation of the system’s response function.

This result confirms the transferability of the calibration approach developed here between the two identical toolboxes realized, indicating the potential scalability of the devices. However, it should be mentioned that the evaluations presented here are still quite preliminary: for a better evaluation of the effective reproducibility, tests should be repeated after prolonged usage, thereby accounting for drift effects, which are expected to decrease the model performance over time. It is presumable that to account for such increasing complexity, the development of suitable calibration transfer logics and algorithms may be required.

## 4. Conclusions

The present work analysed the possibility of using a portable, low-cost sensor toolbox for monitoring methane emissions from landfills, with particular attention to the scalability of the system and to the transferability of the calibration model between replicated units. More specifically, the activity focused on two closely related aspects: verifying whether a model trained on one toolbox can be transferred to a nominally identical replicated unit without the need for a complete individual calibration and assessing the contribution of laboratory data to the predictive performance of the model and to its robustness against the inter-device and inter-session variability that characterises field deployment. These two aspects are particularly relevant in the context of widespread environmental monitoring, where low-cost devices can become truly beneficial only if accompanied by robust, repeatable and economically sustainable calibration strategies. From this perspective, the work does not merely assess the accuracy of the system in a specific case but addresses the broader question of how to move from an experimental prototype to a technology that is replicable and usable on a larger scale.

The experimental work included laboratory calibration campaigns and field walkover campaigns at two active Italian landfills, selected to expose the system to different environmental, meteorological and operational contexts. The two replicated toolboxes V2 and V3 operated in parallel along the same paths, with a portable FID used as the reference instrument and a unified sampling configuration that conveyed the three inlets to the same collection point. The availability, for each campaign, of data acquired simultaneously by the two toolboxes and compared point by point with the FID was a particularly important element of the experimental design, since it allowed the effects due to differences between replicated devices to be clearly distinguished from those associated with the natural variability of the site and of the operating conditions. The inclusion of a second monitoring site also made it possible to assess the generalisability of the model in contexts different from that of initial development.

The results obtained demonstrate that the system is able to fulfil its primary operational objective: in all train → test combinations investigated, the model correctly identifies the emission hotspots along the walkover and reproduces their amplitudes in agreement with the FID measurement, even in the most demanding cross-device configurations. This capability of locating the emission hotspots at the correct position along the walkover path, with an amplitude representative of their magnitude, represents a result of direct operational significance for landfill monitoring practice and supports the hypothesis underlying the scale-up strategy: units replicated according to the same design specifications (same sensors from the same production batch, same electronic and pneumatic architecture, same acquisition logic) exhibit a sufficiently homogeneous response to allow the use of a shared calibration model across nominally identical devices, without the need for a complete individual calibration of each newly produced unit. These considerations will need to be further reinforced through repeated testing over time, since drift is expected to alter the sensor behaviour along with their usage and thus reduce the prediction model performance over time. In order to investigate this aspect in more depth in a realistic usage context, the tests presented here should be repeated after several months of non-simultaneous use of the two toolboxes in real scenarios, in order to also account for the possibility that sensors drift differently depending on the usage context and intensity.

The obtained results show that the model predictions of the two toolboxes are a few seconds delayed compared to the FID measurements. For a preliminary verification and optimisation of the model performances, we adopted a very “rough” approach based on a temporal “shift” of the predicted concentrations: if we shift them onwards by 2 or 6 s, for toolbox V2 and V3, respectively, the agreement between predicted and true concentrations (whereby by true we consider the FID readings) increases considerably to reach R^2^ values of above 0.75 and RMSE of about 250 ppm, which is more than reasonable for the considered application, where the main goal is the detection of hotspots by detecting low concentrations in air and not necessarily achieving high measurement accuracy and precision. The lack of accuracy here is widely compensated by a very low detection threshold: field campaigns proved the ability of these MOX-based toolboxes to detect concentrations in complex environments down to few ppm, which makes the system competitive with more expensive instrumentation, making it useful to detect emission hotspots even through relatively simple ambient air measurements. It is also worth highlighting that the magnitude of the delay, in the order of a few seconds (2 s for V2 and 5–6 s for V3), is entirely compatible with field use considering the typical dynamics of a walkover, in which the operator moves at slow walking speed and the hotspots are traversed over time scales of tens of seconds or minutes.

Nonetheless, the considerations about the “delay” between predicted concentrations and FID measurements are still quite preliminary and will require a deeper investigation in the next future, including a systematic characterization of the different response dynamics of the two measurement systems, a better understanding of the reasons why the delays for the two toolboxes are slightly different (−2 vs. −6), and the evaluation of more refined dynamic compensation models.

Overall, the work demonstrates that a low-cost sensor toolbox can deliver reproducible and transferable performance when developed within a coherent hardware and calibration framework, and that laboratory data play a decisive role in strengthening this transferability and represent a promising tool for the possible development of drift management strategies. These results constitute an important step towards a distributed, scalable methane monitoring strategy that is practically applicable in the field.

Future developments should consider expanding the training and testing datasets, both in terms of the number of tests and of the variety of experimental conditions investigated, in order to verify whether a more extensive database allows further improvements in predictive performance and greater model stability, especially considering long-term use. A second significant line of development will involve the evaluation of alternative machine learning models to XGBoost to assess whether different methods offer advantages in terms of accuracy, robustness or generalisation, particularly in managing inter-device and inter-session variability. In this respect, approaches able to incorporate the temporal dynamics of the sensor response, such as dynamic features or architectures that account for the time evolution of the signals, could also provide a more rigorous treatment of the response delay discussed in Section 3.2.2. Finally, a post-deployment laboratory recalibration of the units was not performed in this work, given the limited operational time accumulated by the units thus far. It will, however, certainly be carried out in the future to assess sensor stability after prolonged field exposure, in particular against H_2_S-induced MOX degradation.

## Figures and Tables

**Table 1:** Cost and characteristics of commercial MOX and NDIR.

Sensor Model	Technology	Supplier	Target Gas	Output Type	Detection Range	Operating RH (%)	Key Features	Approx. Cost (€)
TGS2611-C00 [25]	MOX (analogue)	Figaro Engineering Inc., Osaka, Japan	CH_4_	Analogue (resistance)	50–10,000 ppm (down to <10 ppm reported in the literature)	5 to 95	High sensitivity to CH_4_; no filter; cross-sensitive to VOCs and humidity; heater voltage 5 V; current ~56 mA	~20
TGS2611-E00 [25]	MOX (analogue)	Figaro Engineering Inc., Osaka, Japan	CH_4_	Analogue (resistance)	50–10,000 ppm (down to <10 ppm reported in the literature)	5 to 95	Integrated activated carbon filter reducing but not eliminating cross-sensitivity to interferents (ethanol, H_2_, isobutane); heater voltage 5 V; current ~56 mA	~20
SGP40 [24]	MOX (digital)	Sensirion AG, Stäfa, Switzerland	VOC	Digital (I^2^C)	Ethanol: 0–1000 ppm; CH_4_ sensitivity not specified in the datasheet	0 to 90	On-chip RH and T compensation; SRAW_VOC raw signal output; low power consumption (2.6 mA at 3.3 V); warm-up time ~3 min	~10
MH-441D [19]	NDIR	Winsen Electronics Technology Co., Zhengzhou, China	CH_4_	Analogue (0.4–2.0 V) + Digital (UART)	0–100,000 ppm (effective threshold > 1000 ppm)	0 to 95 (non-condensing)	Selective for CH_4_; O_2_-independent; T90 < 30 s; integrated temperature compensation; ATEX certified (Exib IIb T4 Gb); used as internal reference and alarm trigger	~90

**Table 2:** “Methane only” calibration protocol.

Step	CH_4_ (ppm)	Phase	Duration (s)
1	1	Progressive step-up	600
2	5	Progressive step-up	600
3	10	Progressive step-up	600
4	50	Progressive step-up	600
5	100	Progressive step-up	600
6	500	Progressive step-up	600
7	1000	Progressive step-up	600
8	1	Baseline	600
9	10	Absolute step	600
10	1	Baseline	600
11	50	Absolute step	600
12	1	Baseline	600
13	100	Absolute step	600
14	1	Baseline	600
15	500	Absolute step	600
16	1	Baseline	600
17	1000	Absolute step	600
18	1	Baseline	600

**Table 3:** Calibration protocol with methane and interfering gases (ethanol and acetone).

Step	CH_4_ (ppm)	EtOH/Acetone	Duration (s)
1	0	0	600
2	10	1	600
3	10	2	600
4	10	3	600
5	0	0	600
6	0	2	600
7	0	0	600
8	50	3	600
9	50	5	600
10	50	8	600
11	0	0	600
12	0	5	600
13	0	0	600
14	100	5	600
15	100	10	600
16	100	15	600
17	0	0	600
18	0	10	600
19	0	0	600
20	500	25	600
21	500	50	600
22	500	75	600
23	0	0	600
24	0	50	600
25	0	0	600
26	1000	50	600
27	1000	100	600
28	1000	150	600
29	0	0	600
30	0	100	600
31	0	0	600

**Table 4:** Calibration protocol with methane and interfering gases (CO_2_).

Step	CH_4_ (ppm)	CO_2_	Duration (s)
1	0	0	600
2	10	5	600
3	10	10	600
4	10	20	600
5	0	0	600
6	0	10	600
7	0	0	600
8	50	25	600
9	50	50	600
10	50	100	600
11	0	0	600
12	0	50	600
13	0	0	600
14	100	50	600
15	100	100	600
16	100	200	600
17	0	0	600
18	0	100	600
19	0	0	600
20	500	250	600
21	500	500	600
22	500	750	600
23	0	0	600
24	0	500	600
25	0	0	600
26	1000	500	600
27	1000	1000	600
28	1000	1500	600
29	0	0	600
30	0	1000	600
31	0	0	600

**Table 5:** Summary of the laboratory calibration campaign.

Test Type	Gas	CH_4_ Range (ppm)	Interferent Range (ppm)	RH (%)	No. of Tests
Methane only	CH_4_	1–1000	—	20	2
Methane only	CH_4_	1–1000	—	40	2
Methane only	CH_4_	1–1000	—	60	2
Methane only	CH_4_	1–1000	—	80	2
CH_4_ + CO_2_	CH_4_, CO_2_	10–1000	5–1000	30	1
CH_4_ + CO_2_	CH_4_, CO_2_	10–1000	5–1000	60	1
CH_4_ + ethanol	CH_4_, EtOH	10–1000	1–150	30	1
CH_4_ + ethanol	CH_4_, EtOH	10–1000	1–150	60	1
CH_4_ + acetone	CH_4_, acetone	10–1000	1–150	30	1
CH_4_ + acetone	CH_4_, acetone	10–1000	1–150	60	1

**Table 6:** Test set error metrics (walkover NW3, field-only training) stratified by FID-measured concentration range, toolboxes V2 and V3.

CH_4_ Range	MAE V2	RMSE V2	MAE V3	RMSE V3
0–50 ppm	11.6	69.4	18.2	130.3
50–200 ppm	95.0	410.1	96.3	392.7
>200 ppm	1026.3	1640.1	1258.5	2129.8

**Table 7:** Comparison between lag and model metrics for the V2 → V2 configuration (training including laboratory data).

Lag	R^2^	MAE	RMSE
−6	0.409	64.76	404.16
−5	0.545	56.26	354.28
−4	0.665	50.75	304.11
−3	0.736	48.87	270.09
−2	0.749	51.54	263.19
−1	0.625	61.09	321.56
0	0.469	71.23	382.86
1	0.282	80.12	445.03
2	0.109	88.46	495.97
3	−0.057	95.89	540.25

**Table 8:** Comparison between lag and model metrics for the V3 → V3 configuration (training including laboratory data).

Lag	R^2^	MAE	RMSE
−6	0.773	51.22	252.44
−5	0.761	51.62	259.21
−4	0.679	55.53	291.37
−3	0.55	63.99	355.82
−2	0.365	74.18	422.37
−1	0.168	83.87	483.56
0	−0.01	91.1	532.87
1	−0.132	96.48	564.12
2	−0.228	101.49	587.49
3	−0.27	105.08	597.66

## Data Availability

The data presented in this study are available on request from the corresponding author.

## Supplementary Materials

The following supporting information can be downloaded at: https://www.mdpi.com/article/10.3390/s26134321/s1, Figure S1: Temperature (first) and relative humidity (second) measured by the SHT40 inside the chamber of toolbox V2 during a methane calibration test (Table 2); Figure S2: Temperature (a) and relative humidity (b) measured by the SHT40 inside the chamber of toolbox V3 during a methane calibration test (Table 2); Figure S3: Raw response of the TGS2611-C00 (first) and TGS2611-E00 (second) sen-sors of toolbox V2 during the methane calibration test (Table 2); Figure S4: Response of the MH-441D NDIR sensor (left axis) compared with the CH_4_ concentration set-point (right axis) during the methane calibration test of toolbox V2 (Table 2); Figure S5: Raw response of the TGS2611-C00 (first) and TGS2611-E00 (second) sen-sors of toolbox V3 during the methane calibration test (Table 2); Figure S6: Response of the TGS2611-C00 (first) and TGS2611-E00 (second) sensors of toolbox V2 to the acetone interferent test (Table 3); Figure S7: Response of the TGS2611-C00 (first) and TGS2611-E00 (second) sensors of toolbox V3 to the acetone interferent test (Table 3); Figure S8: Response of the TGS2611-C00 (first) and TGS2611-E00 (second) sensors of toolbox V2 to the ethanol interferent test (Table 3); Figure S9: Response of the TGS2611-C00 (first) and TGS2611-E00 (second) sensors of toolbox V3 to the ethanol interferent test (Table 3); Figure S10: Response of the TGS2611-C00 (first) and TGS2611-E00 (second) sensors of toolbox V2 to the carbon dioxide interferent test (Table 4); Figure S11: Response of the TGS2611-C00 (first) and TGS2611-E00 (second) sensors of toolbox V3 to the carbon dioxide interferent test (Table 4).
