# Supplementary material for: Measuring Methane Emissions in Ambient Air with a Low-Cost, Portable Sensor System: Focus on Scalability and Transferability of the Model
Source: Sensors (Basel). 2026 Jul 7;26(13):4321. doi: 10.3390/s26134321 (PMC13364052; doi:10.3390/s26134321)
Supplement: Supplementary file 1 [file sensors-26-04321-s001.zip › sensors-4368611-supplementary.pdf]

sensors

MDPI

Supplementary Materials

Measuring Methane Emissions in Ambient Air with a Low-Cost, Portable Sensor System: Focus on Scalability and Transferability of the Model

Lorenzo Bertin [1,*], Matteo Mentasti [2], Fabrizio Pittorino [2], Veronica Villa [1], Emanuele Zanni [2], Gabriele Viscardi [2], Yuri Ponzani [3], Andrea Massara [4], Manuel Roveri [2], Raffaele Dellaca' [2] and Laura Capelli [1]

1 Department of Chemistry, Materials and Chemical Engineering "Giulio Natta", Politecnico di Milano, Piazza Leonardo da Vinci 32, 20133 Milan, Italy; veronica.villa@polimi.it (V.V.); laura.capelli@polimi.it (L.C.)
2 Department of Electronics, Information, and Bioengineering, Politecnico di Milano, via Giuseppe Ponzio 34, 20133 Milan, Italy; matteo.mentasti@polimi.it (M.M.); fabrizio.pittorino@polimi.it (F.P.); emanuele.zanni@polimi.it (E.Z.); gabriele.viscardi@polimi.it (G.V.); manuel.roveri@polimi.it (M.R.); raffaele.dellaca@polimi.it (R.D.)
3 Recycle2Trade Ltd, Science Park Square, Brighton BN1 9SB, UK; info@recycle2trade.com
4 Integraciones Digitales Gold SL (Indigo), 8820 Barcelona, Spain; andrea@adaptivecity.com
* Correspondence: lorenzo.bertin@polimi.it

Supplementary Materials

This document collects the additional figures referenced in the manuscript. They support the laboratory characterisation of the two replicated toolboxes (V2 and V3). All table references (Tables 2–4) refer to the main article. Unless otherwise stated, the methane-only tests follow the step protocol of Table 2 (CH4 from 1 to 1000 ppm, 600 s per step), while the interferent tests follow the protocols of Table 3 (acetone and ethanol) and Table 4 (CO2).

S1. Temperature and Relative Humidity Inside the Measurement Chamber

During the laboratory tests, the temperature and relative humidity inside each toolbox chamber were continuously monitored by the SHT40 sensor while all sensors were operating simultaneously. Figures S1 and S2 report these quantities for toolboxes V2 and V3, recorded during a methane calibration test (Table 2).

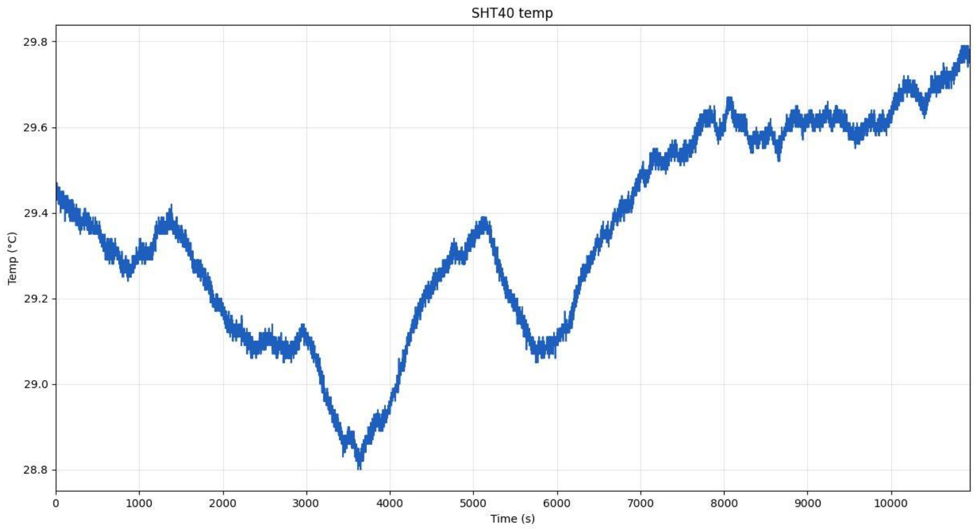

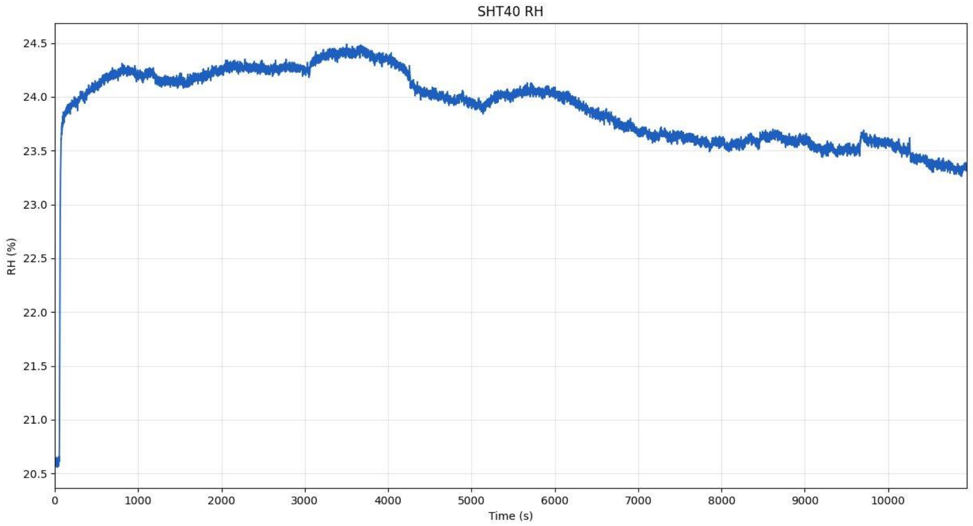

Figure S1. Temperature (first) and relative humidity (second) measured by the SHT40 inside the chamber of toolbox V2 during a methane calibration test (Table 2).

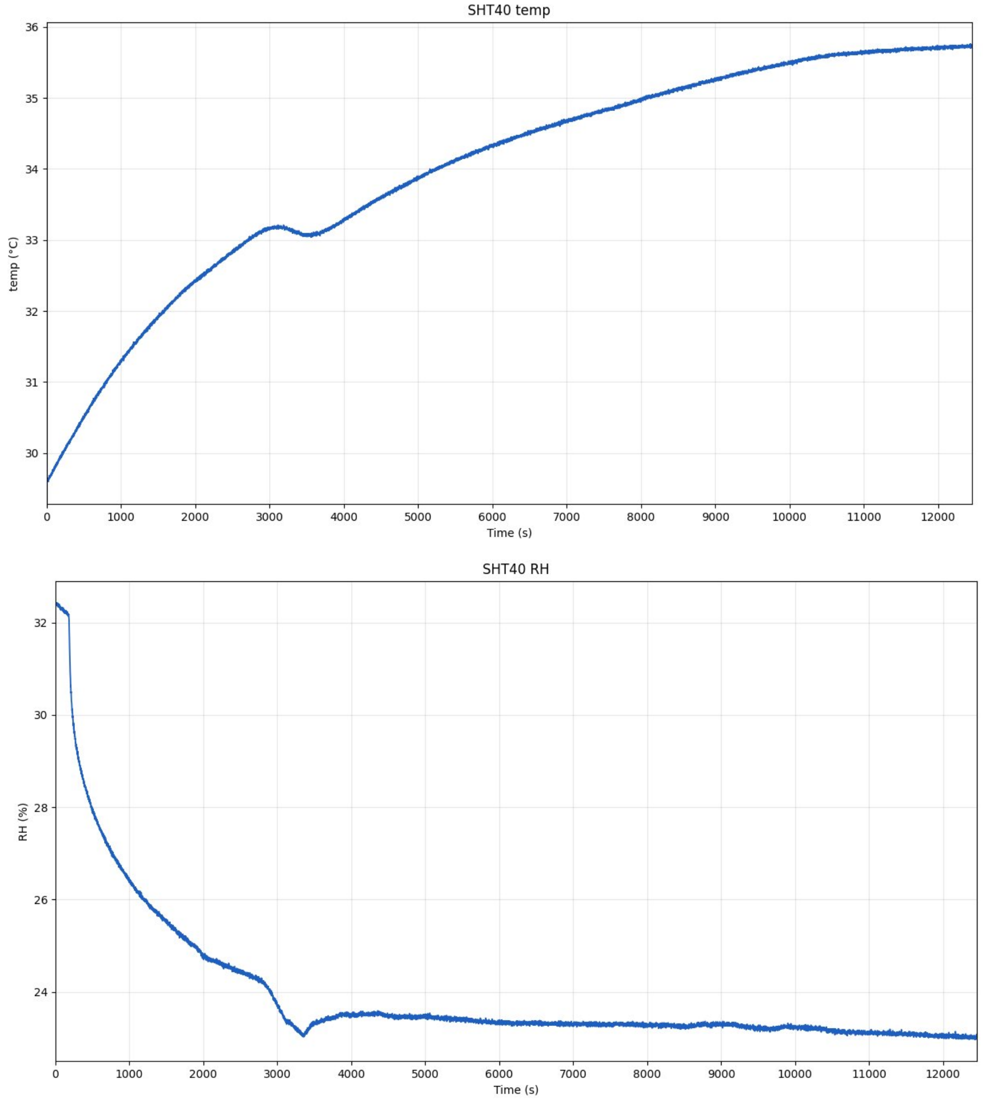

Figure S2. Temperature (a) and relative humidity (b) measured by the SHT40 inside the chamber of toolbox V3 during a methane calibration test (Table 2).

S2. Sensor Response During the Methane Calibration Test

Figures S3 to S5 show the raw response of the sensors during the methane step protocol of Table 2. For both toolboxes, the two TGS2611 units reproduce the concentration steps with fast response and recovery and a stable baseline between steps, while the MH-441D NDIR sensor responds appreciably only to the highest concentration steps (≈600–1000 ppm).

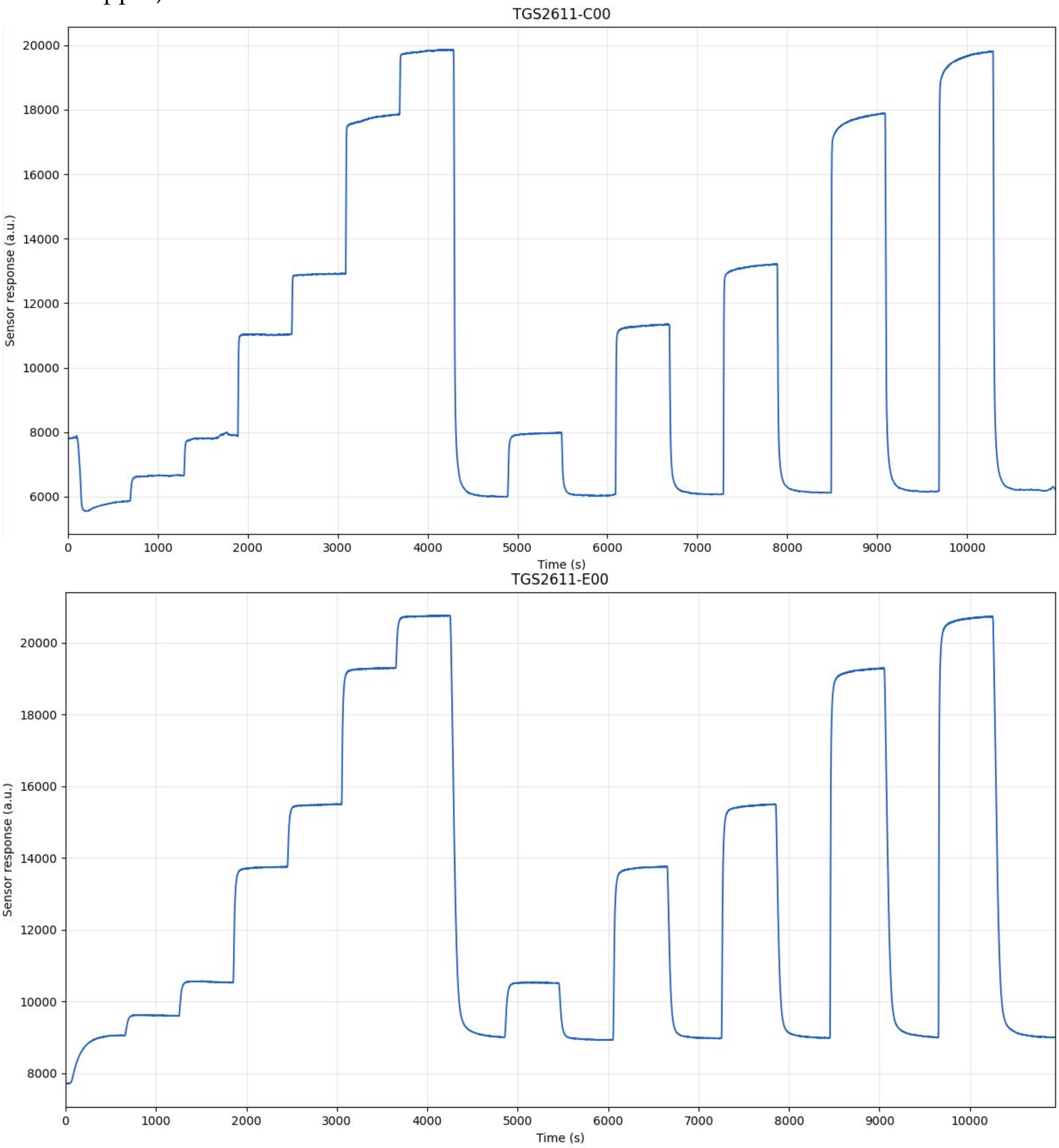

Figure S3. Raw response of the TGS2611-C00 (first) and TGS2611-E00 (second) sensors of toolbox V2 during the methane calibration test (Table 2).

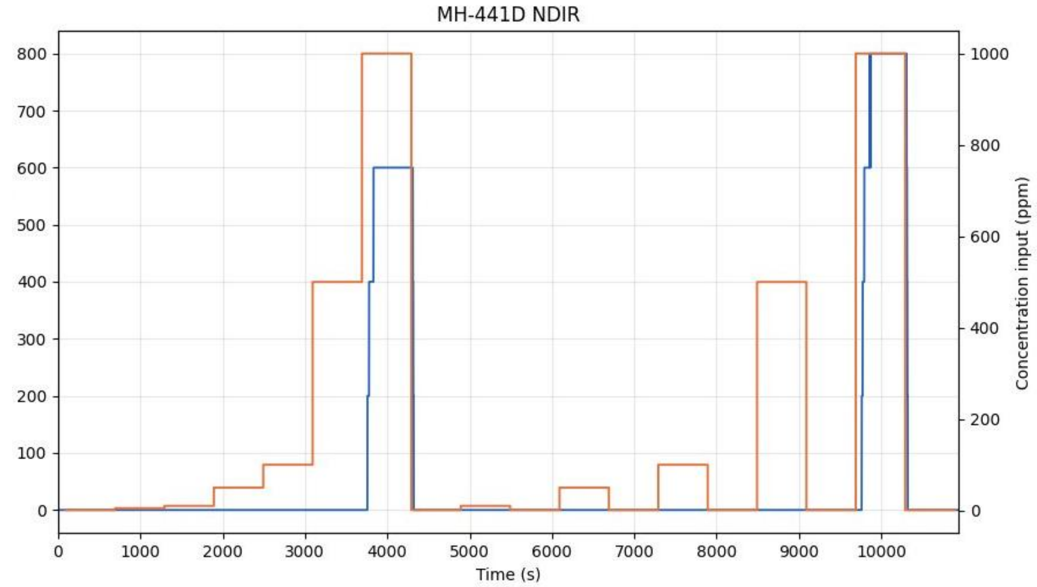

Figure S4. Response of the MH-441D NDIR sensor (left axis) compared with the CH_4 concentration set-point (right axis) during the methane calibration test of toolbox V2 (Table 2).

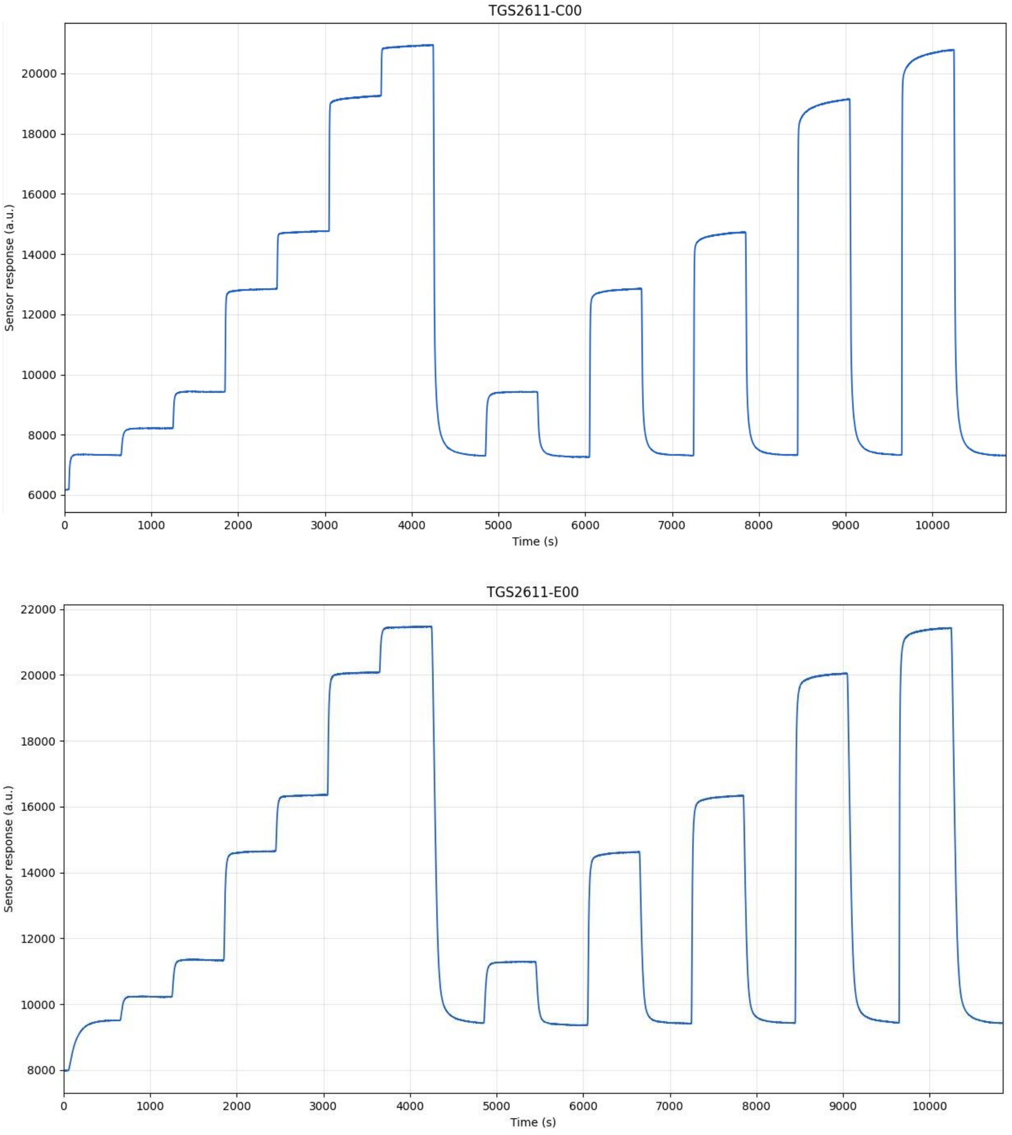

Figure S5. Raw response of the TGS2611-C00 (first) and TGS2611-E00 (second) sensors of toolbox V3 during the methane calibration test (Table 2).

S3. Cross-sensitivity of the MOX Sensors to Interferents

To illustrate the residual cross-sensitivity of the MOX sensors discussed in Section 2.1, Figures S6 to S11 show the response of the two TGS2611 sensors to the interferent tests carried out with acetone and ethanol (Table 3) and with carbon dioxide (Table 4).

Acetone

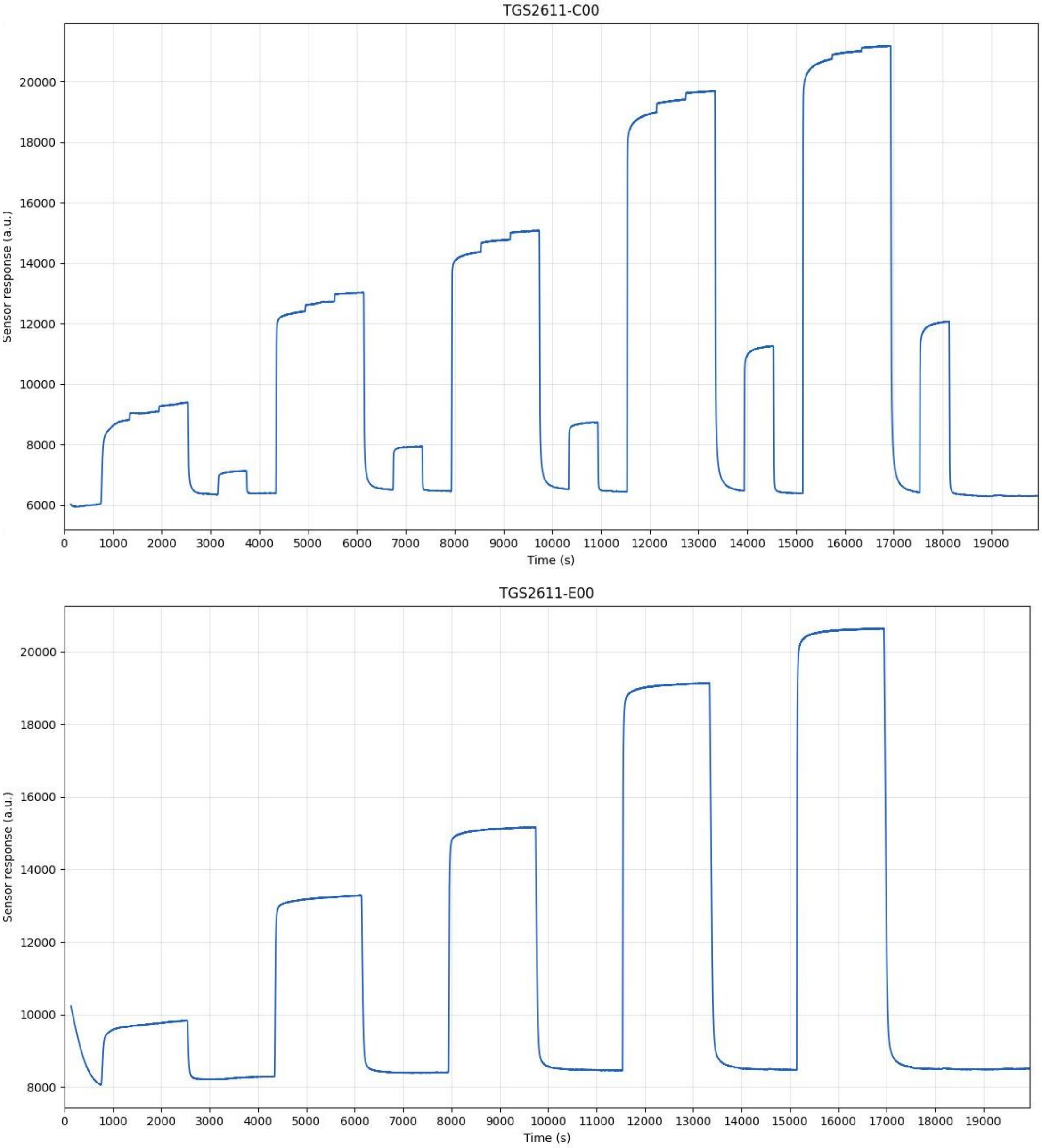

Figure S6. Response of the TGS2611-C00 (first) and TGS2611-E00 (second) sensors of toolbox V2 to the acetone interferent test (Table 3).

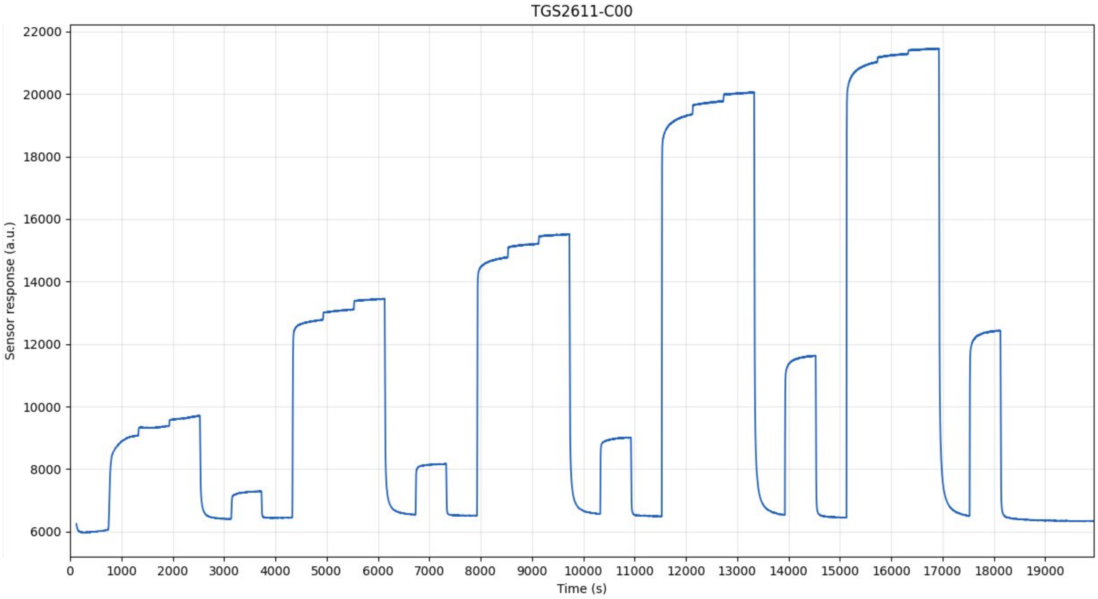

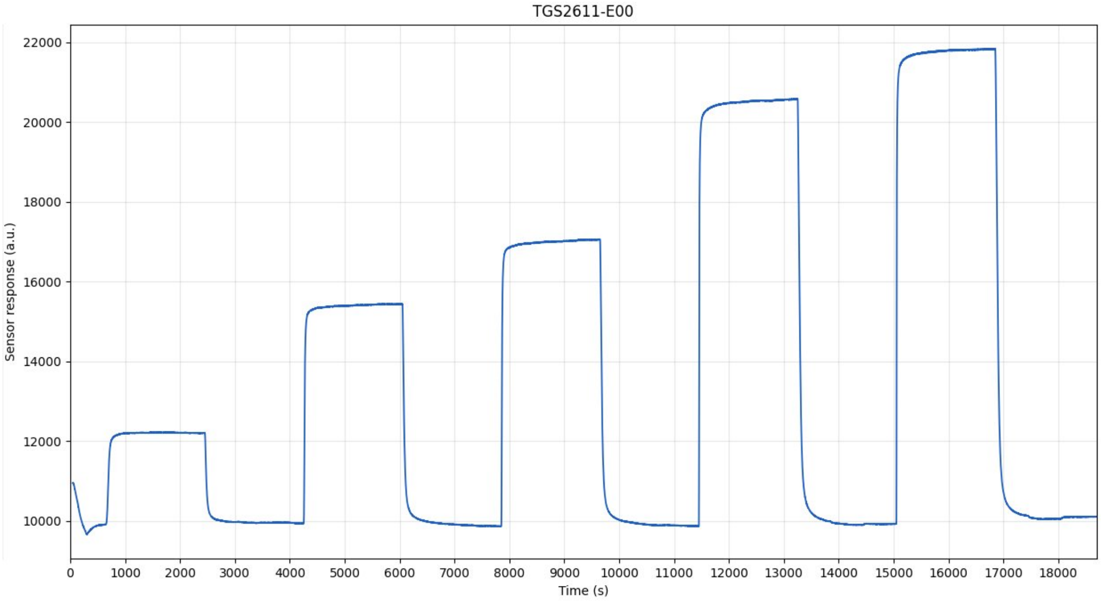

Figure S7. Response of the TGS2611-C00 (first) and TGS2611-E00 (second) sensors of toolbox V3 to the acetone interferent test (Table 3).

Ethanol

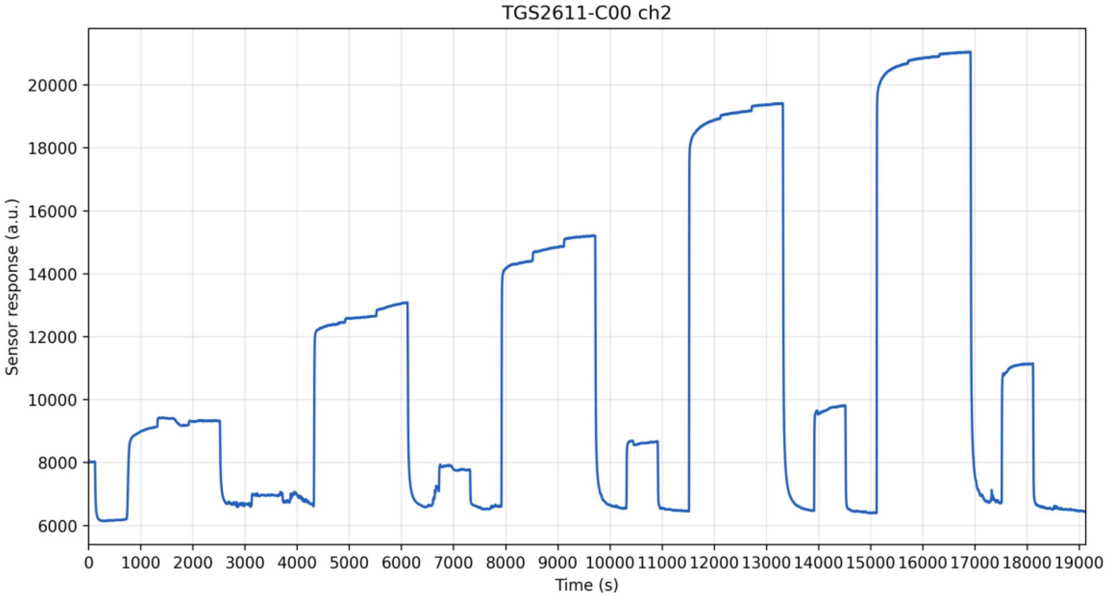

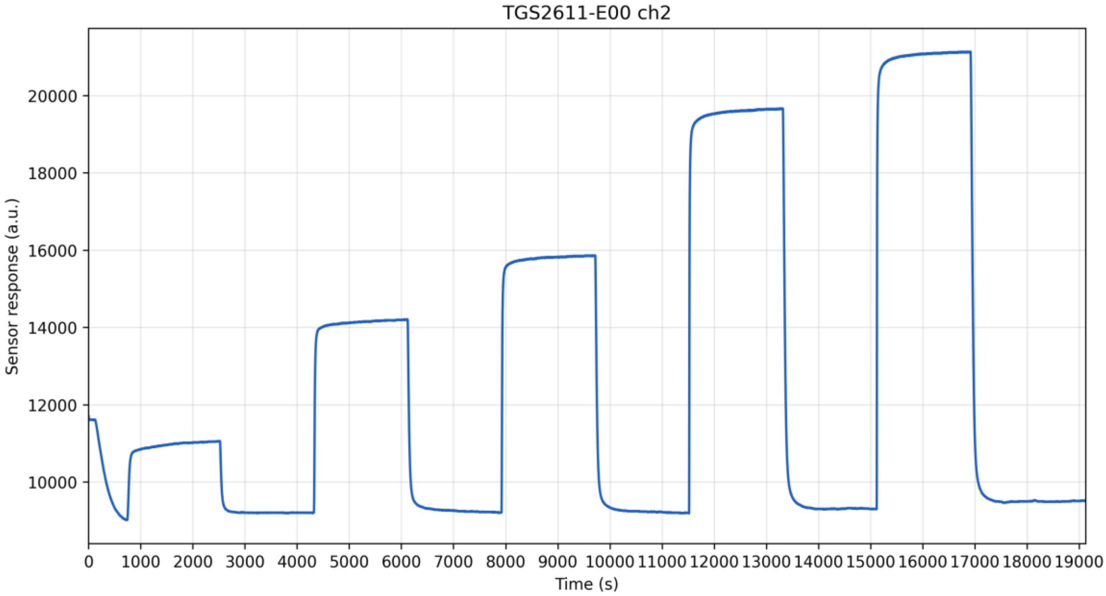

Figure S8. Response of the TGS2611-C00 (first) and TGS2611-E00 (second) sensors of toolbox V2 to the ethanol interferent test (Table 3).

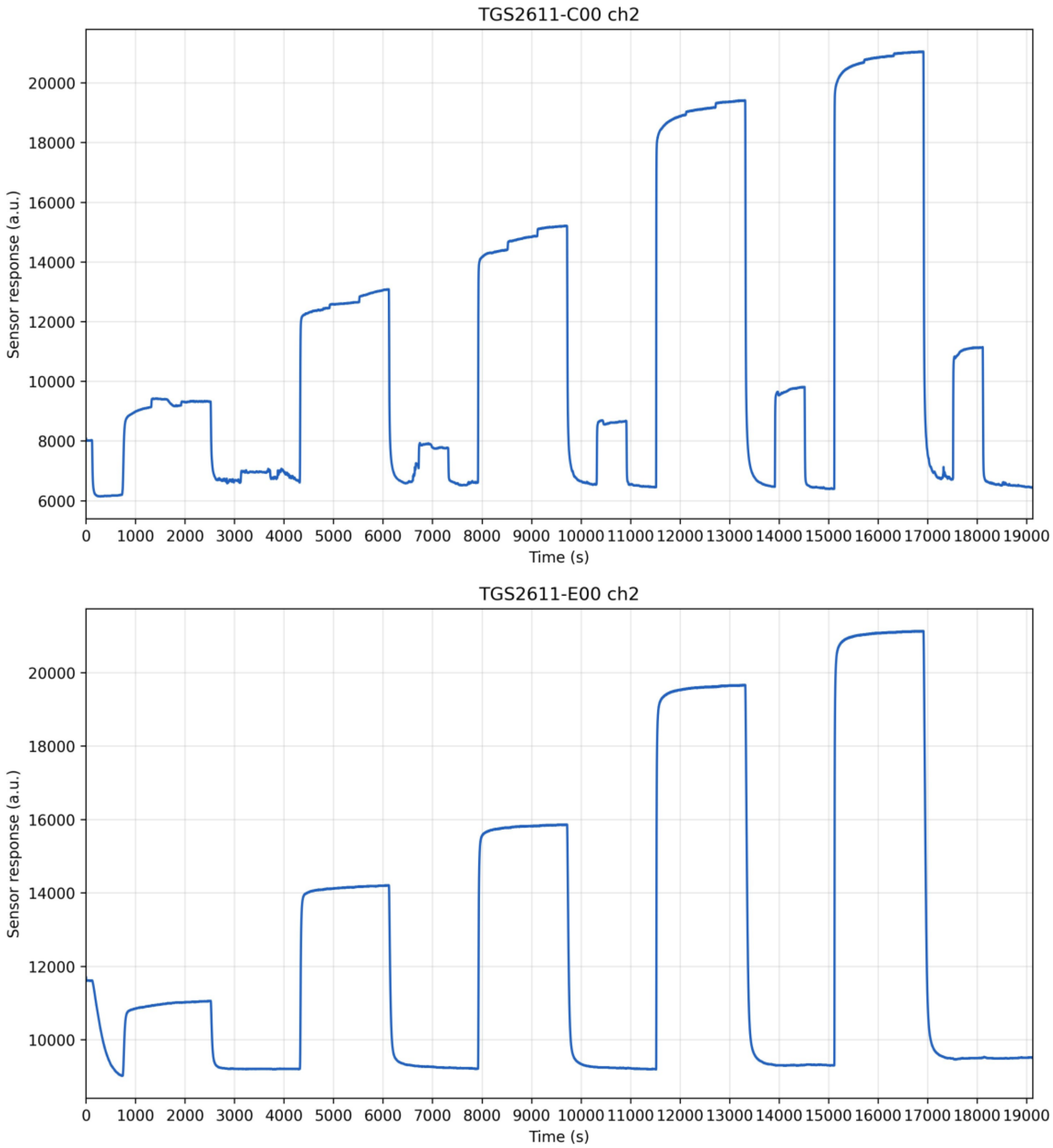

Figure S9. Response of the TGS2611-C00 (first) and TGS2611-E00 (second) sensors of toolbox V3 to the ethanol interferent test (Table 3).

Carbon dioxide

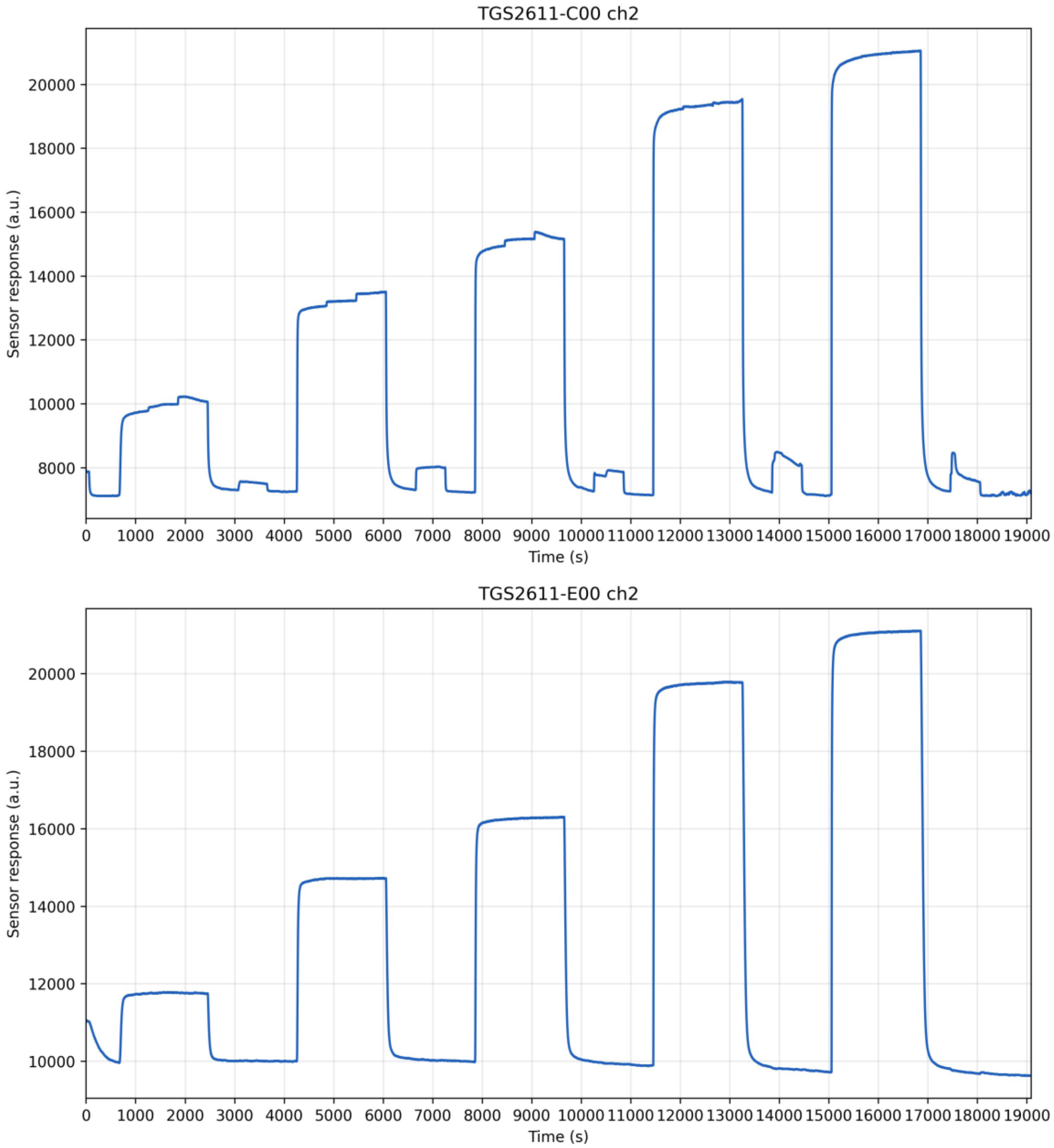

Figure S10. Response of the TGS2611-C00 (first) and TGS2611-E00 (second) sensors of toolbox V2 to the carbon dioxide interferent test (Table 4).

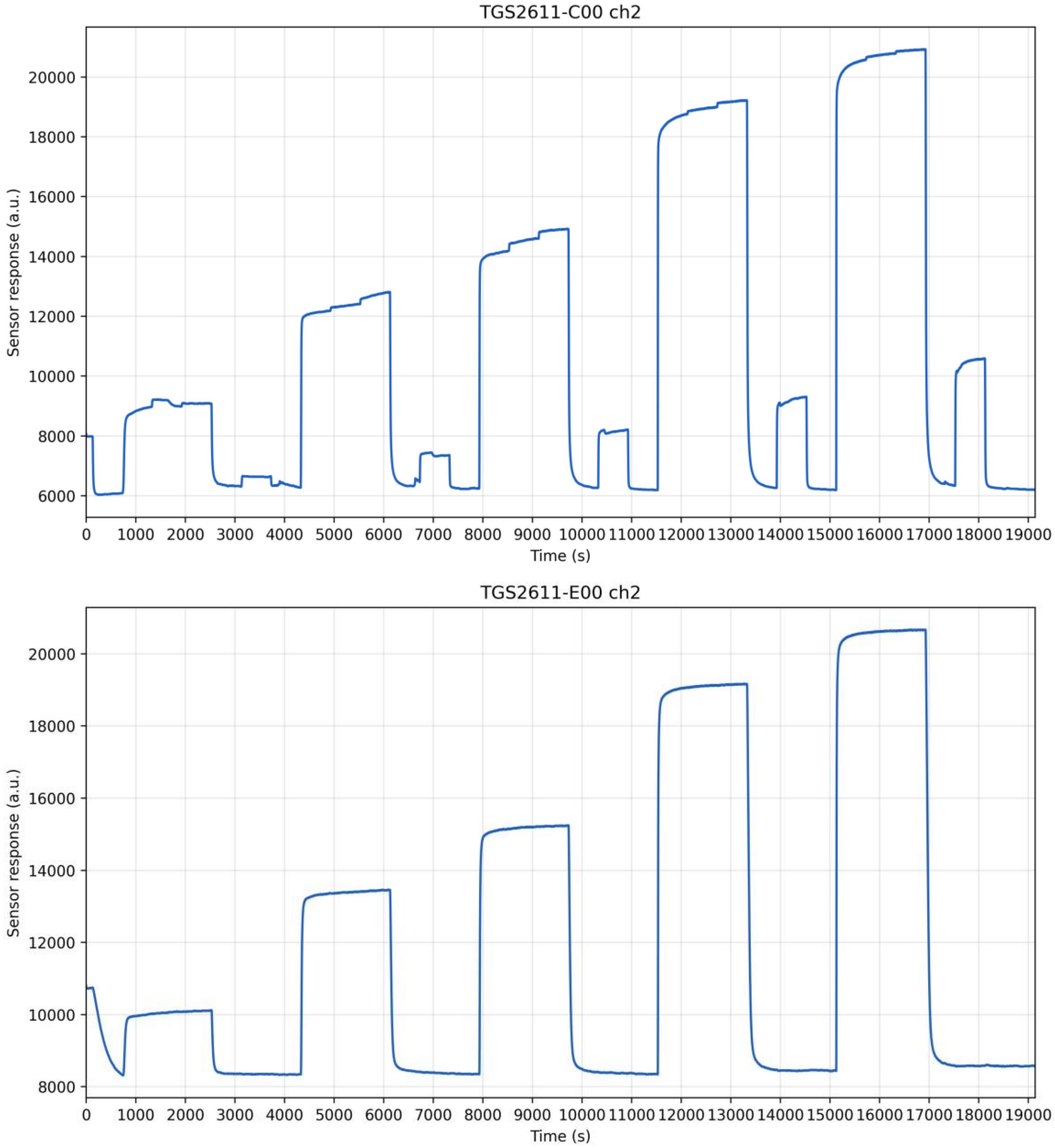

Figure S11. Response of the TGS2611-C00 (first) and TGS2611-E00 (second) sensors of toolbox V3 to the carbon dioxide interferent test (Table 4).
